# Microglia regulate GABAergic neurogenesis in prenatal human brain through IGF1

**DOI:** 10.1038/s41586-025-09362-8

**Published:** 2025-08-06

**Authors:** Diankun Yu, Samhita Jain, Andi Wangzhou, Beika Zhu, Wenyuan Shao, Elena J. Coley-O’Rourke, Stacy De Florencio, JaeYeon Kim, Jennifer Ja-Yoon Choi, Mercedes F. Paredes, Tomasz J. Nowakowski, Eric J. Huang, Xianhua Piao

**Affiliations:** 1 Weill Institute for Neurosciences, University of California San Francisco, San Francisco, CA USA; 2 Division of Neonatology, Department of Pediatrics, University of California San Francisco, San Francisco, CA USA; 3 Eli and Edythe Broad Center of Regeneration Medicine and Stem Cell Research, University of California San Francisco, San Francisco, CA USA; 4 Department of Neurology, University of California San Francisco, San Francisco, CA USA; 5 Department of Pathology, University of California San Francisco, San Francisco, CA USA; 6 Department of Neurological Surgery, University of California San Francisco, San Francisco, CA USA; 7 Department of Anatomy, University of California San Francisco, San Francisco, CA USA; 8 Department of Psychiatry and Behavioral Sciences, University of California San Francisco, San Francisco, CA USA; 9 Pathology Service, San Francisco VA Health Care System, San Francisco, CA USA; 10 Newborn Brain Research Institute, University of California San Francisco, San Francisco, CA USA; 11 Present Address: Department of Pathology & Immunology, Washington University School of Medicine, St. Louis, MO USA

**Keywords:** Neural progenitors, Neuroimmunology, Neuronal development, Microglia, Developmental neurogenesis

## Abstract

GABAergic neurons are essential cellular components of neural circuits. Their abundance and diversity have increased significantly in the human brain, contributing to the expanded cognitive capacity of humans^1^. However, the developmental mechanism underlying the extended production of GABAergic neurons in the human brain remains elusive. Here we uncovered the microglial regulation of the sustained proliferation of GABAergic progenitors and neuroblasts in the human medial ganglionic eminence (hMGE). We showed that microglia are preferentially distributed in the proliferating zone and identified insulin-like growth factor 1 (IGF1) and its receptor IGR1R as the predicted top ligand–receptor pair underlying microglia–progenitor communication in the prenatal hMGE. Using our newly developed neuroimmune hMGE organoids, which mimic the hMGE cytoarchitecture and developmental trajectory, we demonstrated that microglia-derived IGF1 promotes progenitor proliferation and production of GABAergic neurons. Conversely, IGF1-neutralizing antibodies and *IGF1* knockout human embryonic stem-cell-induced microglia abolish the induced microglia-mediated progenitor proliferation. Together, these findings revealed a previously unappreciated role of microglia-derived IGF1 in promoting the proliferation of neural progenitors and the development of GABAergic neurons in the human brain.

## Abstract (web summary)

Microglia-derived IGF1 promotes the proliferation of GABAergic progenitors and neuroblasts in the human medial ganglionic eminence, contributing to the extended production of GABAergic neurons and an expanded cognitive capacity in the human brain.

## Main

In the adult human neocortex, about 25–50% of cortical neurons are γ-aminobutyric acid-containing (GABAergic) inhibitory interneurons^1–4^. They serve as the principal sources of cortical inhibitory input and have a crucial role in maintaining excitation–inhibition balance and functional rhythms in the brain^5,6^. Disruptions in interneuron number and function have been implicated in various neurological and psychiatric disorders, including autism spectrum disorder^7,8^, epilepsy^9^ and schizophrenia^10^. In the prenatal human brain, GABAergic interneurons are generated in ganglionic eminences of the ventral telencephalon^11–13^. The medial ganglionic eminence (MGE) gives rise to most parvalbumin-positive (PV^+^) and somatostatin-positive (SST^+^) cortical interneurons^7^. Human MGE (hMGE) possesses several unique features that are distinct from those of other species to meet the needs of a drastically expanded cerebral cortex. First, neurogenesis in hMGE is most active during the second and third trimesters^14^. This active neurogenesis is followed by extensive migration of GABAergic neurons around the periventricular zone in the neonatal stage for at least 6 months postnatally^15^. Second, young GABAergic neuroblasts are organized as DCX^+^ cell-enriched nests (DENs) that have sustained proliferation up to the early postnatal stage. GABAergic neuroblasts within DENs are surrounded by NESTIN^+^ and SOX2^+^ radial glia that extend from the ventricular zone and inner subventricular zone (iSVZ) to the outer subventricular zone (oSVZ)^11,14^. Third, the DCX^+^SOX2^+^ neuroblasts inside DENs exhibit regional differences in their proliferation potentials, with those at the edge of DENs showing a higher Ki-67 labelling index^14^. Although these observations support the notion that DCX^+^ neuroblasts in DENs have the capacity to undergo sustained neurogenesis in hMGE^11,14^, the external environmental cues that regulate hMGE neurogenesis remain largely unclear.

Microglia, which are specialized tissue-resident macrophages in the central nervous system, are the primary immune cells in the brain parenchyma. Originating from the yolk sac and entering the brain during early gestation^16^, microglia have been shown to be involved in brain development, including neurogenesis^17^, angiogenesis^18,19^, neuronal survival^20^, myelination^21–24^, synaptogenesis^25^ and synaptic pruning^26–30^. It has been shown that microglia regulate PV^+^ interneuron development and positioning^31^ and lead to PV^+^ interneuron deficits in setting of maternal immune activation in the mouse brain^32^. However, it remains unclear whether and how microglia affect the development of GABAergic neurons in the human brain. Here we show that during the late second trimester, the iSVZ and oSVZ of hMGE are populated with microglia that are near the radial glia and Ki-67^+^ proliferating neuroblasts at the periphery of DENs. Single-nucleus transcriptomic studies of human brains from the late second trimester to the early postnatal stage uncovered insulin-like growth factor 1 (IGF1) and its receptor IGF1R as the top candidate pathways in mediating the communication between microglia and interneuron progenitors. Further studies using human MGE neuroimmune organoids (MGEOs), in which human embryonic stem-cell (hESC)-induced microglia (iMG) were transplanted into human pluripotent stem-cell (hPSC)-derived MGE organoids, support that the preferential accumulation of microglia in subventricular zone (SVZ) is related to progenitor proliferation, and that microglia-derived IGF1 promotes progenitor proliferation and interneuron production.

## Microglia distribution in hMGE

**Fig. 1:**
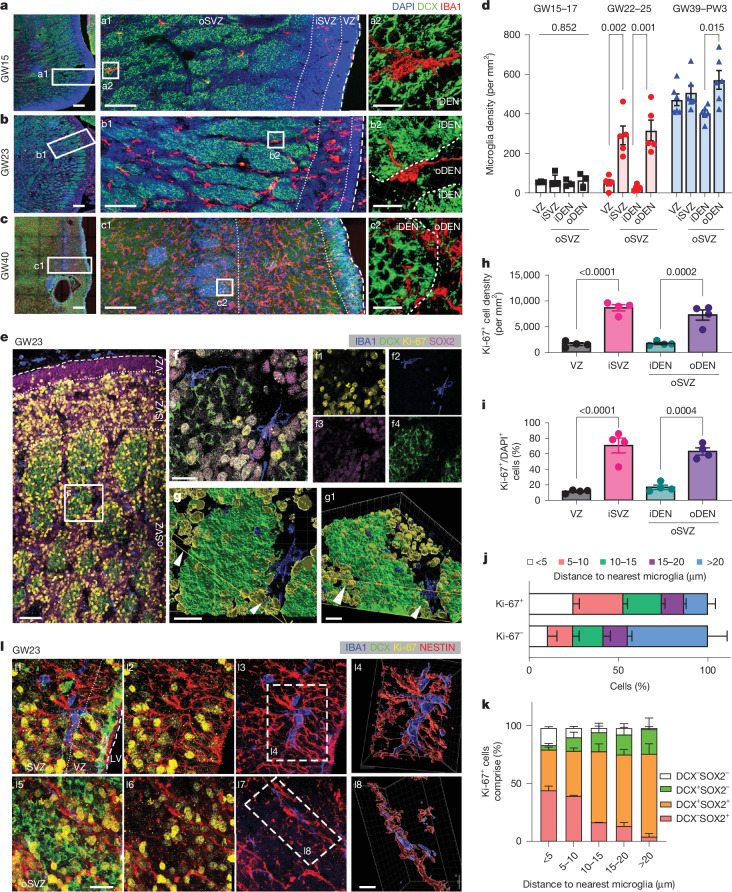
Microglia are in proximity to MGE progenitors in the developing human brain. **a**–**d**, IHC images (**a**–**c**) and bar graphs (**d**) showing microglia in the developing hMGE at GW15 (**a**,**d**), GW23 (**b**,**d**) and GW40 (**c**,**d**). Microglia were highly concentrated in the iSVZ and oDENs of oSVZ at GW22–25. **e**,**f**, IHC images showing the spatial relationship between Ki-67^+^ proliferating progenitors and microglia in the GW23 hMGE sections; **f** provides a magnified view of the region indicated in **e**. **g**, Three-dimensional reconstruction revealing the close proximity between microglia and Ki-67^+^ proliferating progenitors in the oSVZ of GW23 hMGE. Arrowheads point to the Ki-67^+^ clusters near the microglia in oSVZ. **h**,**i**, Bar graphs showing the density (**h**) and percentage (**i**) of Ki-67^+^ progenitors in GW22–25 hMGE. **j**, Percentage of Ki-67^+^ and Ki-67^−^ cells as their distance to microglia increased in the oSVZ of hMGE. **k**, Cell composition analysis of Ki-67^+^ progenitors showing Ki-67^+^ cells in proximity to microglia comprising SOX2^+^DCX^−^ radial glia and SOX2^+^DCX^+^ neuroblasts. **l**, IHC images and 3D reconstruction indicating that microglia closely contacted NESTIN^+^ projections in GW23 hMGE. For statistics, *n* (biological repeats) = 3, 5 and 6 (**d**); *n* = 4 (**h**,**i**); *n* = 4 (**j**,**k**). One-way analysis of variance (ANOVA) and post hoc Bonferroni’s test for **d**,**h** and **i**; two-way repeated measures ANOVA for **j** and **k** showed significant interaction effects (*P* = 0.006 (**j**) and *P* < 0.0001 (**k**)). Data in **d** and **h**–**k** are shown as mean ± s.e.m. Scale bars, 200 μm (**a**–**c** (left)), 100 μm (**a**–**c** (middle)), 10 μm (**a**–**c** (right),**l** (right)), 50 μm (**e**), 20 μm (**f**,**g**,**l** (left)).

To study microglial function in hMGE development, we first surveyed the temporal and spatial distributions in the hMGE of neurotypical human postmortem brains from gestational week (GW) 15 to postnatal week 3 using IBA1 immunohistochemistry (IHC) (Fig. 1a–d). At GW15–17, microglia were sparsely sprinkled throughout hMGE (Fig. 1a,d). However, by GW22–25, there was a clear locational preference, with most microglia seen in iSVZ and oSVZ (Fig. 1b,d). Microglia in oSVZ accumulated in the area outside of DENs (oDENs) and encased the outer rim of DENs, with rare microglial processes extending into the inside of DENs (Fig. 1b,d). By GW39 to postnatal week 3, the density of microglia in the iSVZ and oSVZ of hMGE remained high, and more microglial processes could be identified in the inside of DENs than those in the late second trimester (Fig. 1c,d).

To further examine the spatial relationship between microglia and proliferating cells in hMGE during GW22–25, we performed a combined IHC with IBA1 and Ki-67, a marker for cell proliferation. We observed that microglia mostly resided in regions with more abundant proliferative progenitors (Fig. 1e–i). Specifically, many microglia were embedded within the Ki-67^+^ proliferating progenitors in the iSVZ and oDENs of oSVZ (Fig. 1e,f). Through three-dimensional (3D) reconstruction of DENs using IMARIS software, we found that Ki-67^+^ cells were largely clustered around the microglia on the outside and border of DENs, with a gradual decrease in the ratio of Ki-67^+^ cells to Ki-67^−^ cells as the distance to the microglia increased (Fig. 1g,j). Furthermore, Ki-67^+^ progenitors in proximity to microglia were largely SOX2^+^ progenitors, including SOX2^+^DCX^−^ radial glia and SOX2^+^DCX^+^ neuroblasts (Fig. 1e–g,k). To further document the spatial relationship between microglia and MGE progenitors, we performed IHC of IBA1, DCX, NESTIN and Ki-67. We found that microglia were in close contact with and often followed along NESTIN^+^ radial glial fibres (Fig. 1l). In iSVZ, the microglia were mostly ramified and intermingled with several NESTIN^+^ fibres (Fig. 1l(1–4)). Most microglia in oSVZ exhibited polarized morphology along the NESTIN^+^ fibres, with their processes largely aligning and in close contact with those fibres (Fig. 1l(5–8)).

**Extended Data Fig. 1:**
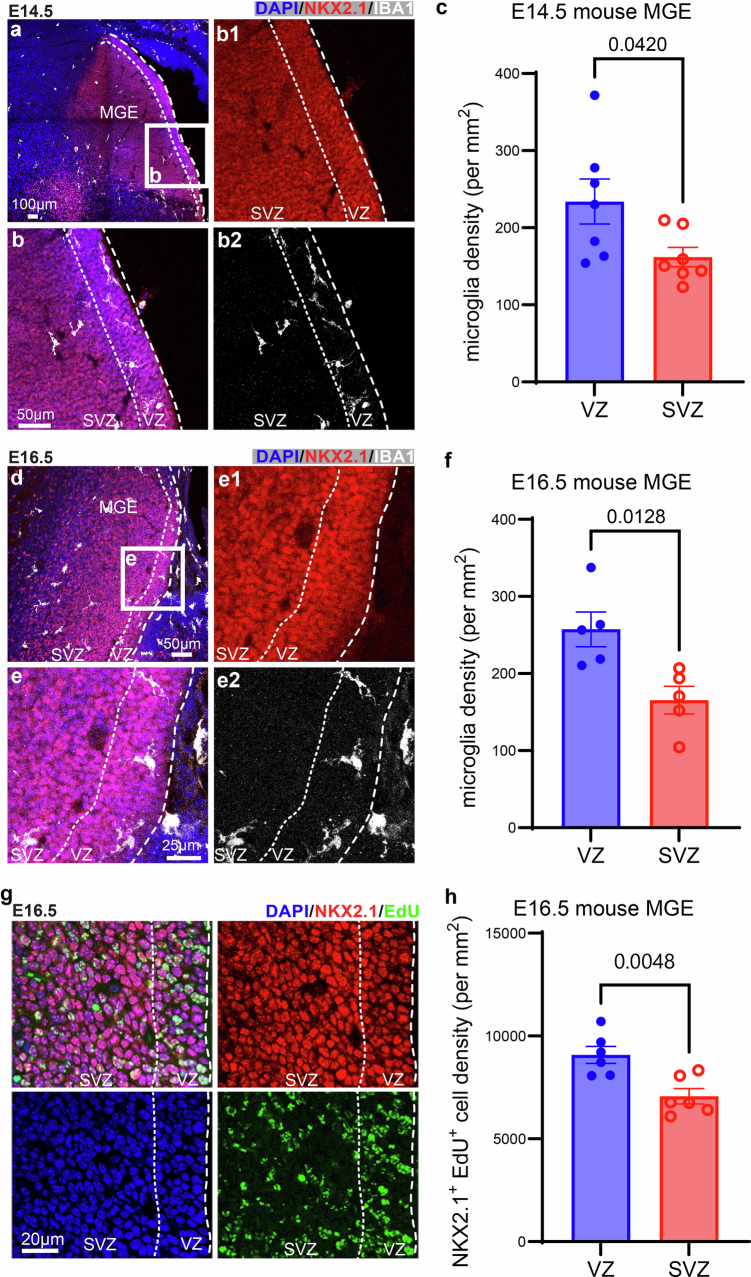
The distribution of microglia and proliferation progenitors in mouse MGE. **a-b**, IHC images showing the distribution of IBA1^+^ microglia in embryonic day (E)14.5 mouse MGE. MGE were labelled by NKX2.1. **c**, Bar graph showing the density of IBA1^+^ microglia is significantly higher in the VZ of E14.5 mouse MGE. **d-e**, IHC images showing the distribution of IBA1^+^ microglia in E16.5 mouse MGE. **f**, Bar graph showing the density of IBA1^+^ microglia is significantly higher in the VZ of E16.5 mouse MGE. **g**, IHC images showing the distribution of EdU^+^NKX2.1^+^ active progenitors in the E16.5 mouse MGE with EdU injected at E14.5. **h**, Bar graph showing the density of NKX2.1^+^EdU^+^ active progenitors is significantly higher in the VZ of E16.5 mouse MGE with EdU injected at E14.5. N = 7, 5, 6 for (c), (f), (h), respectively; unpaired two-tailed t-test. Data are shown as means ± SEM. Source data

Distinct from what was observed in hMGE, the density of microglia was significantly higher in the ventricular zone of mouse MGE on embryonic days 14.5 (Extended Data Fig. 1a–c) and 16.5 (Extended Data Fig. 1d–f), coinciding with the higher density of active proliferating progenitors in the ventricular zone of mouse MGE (Extended Data Fig. 1g,h). This contrast between mouse MGE and hMGE highlights a potential species-specific mechanism for interneuron development.

## Cell communication in the developing human brain

**Fig. 2:**
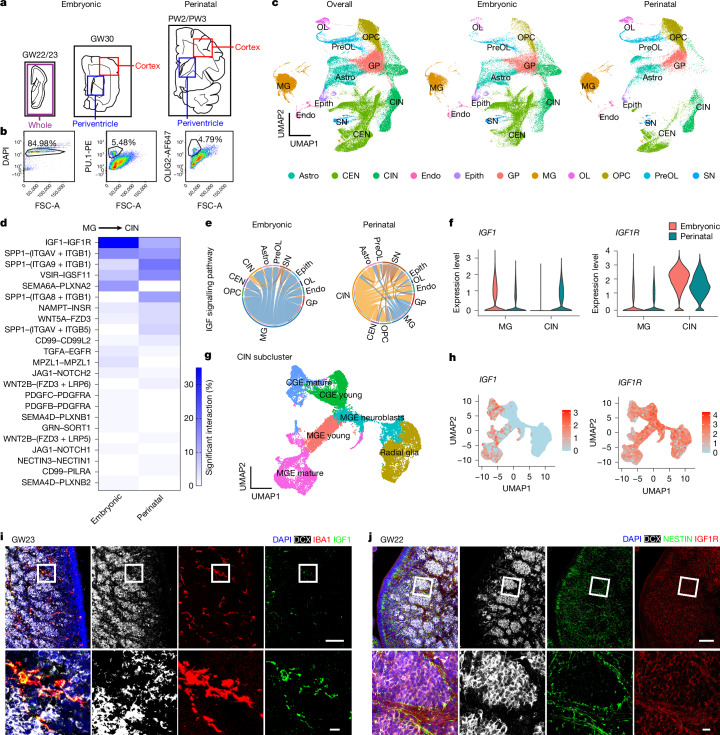
Transcriptomic profiling and cell–cell interaction analysis revealed IGF1–IGF1R as the top potential signalling pathway mediating the communication between interneuron progenitors and microglia in developing hMGE. **a**, Diagrams showing the brain regions used for the snRNA-seq experiment. **b**, Flow cytometry strategies for unenriched DAPI^+^ nuclei and enrichment of PU.1^+^ and OLIG2^+^ nuclei. **c**, UMAP plots showing the 11 identified cell types. **d**, Heat-map plot showing the significant ligand–receptor pairs that potentially mediate microglial regulation of interneuron development at different developmental stages. **e**, Chord plots of IGF signalling pathways at the embryonic and perinatal stages. **f**, Violin plots of *IGF1* and *IGF1R* expression by microglia and interneurons at the embryonic and perinatal stages. **g**, UMAP plots of the subtypes of cortical GABAergic interneurons, including radial glia, MGE neuroblasts, MGE-derived young interneurons (MGE young), MGE-derived mature interneurons (MGE mature), CGE-derived young interneurons (CGE young) and CGE-derived mature interneurons (CGE mature). **h**, Feature plots showing the expression patterns of *IGF1* and *IGF1R* in the interneuron subtypes. **i**, Representative IHC from three biological repeats showing the specific expression of IGF1 in microglia in developing hMGE. **j**, Representative IHC from three biological repeats showing the expression pattern of IGF1R in the developing hMGE. Astro, astrocytes; CEN, cortical excitatory neurons; CIN, cortical GABAergic interneurons; Endo, endothelial cells; Epith, epithelial cells; GP, glia progenitors; MG, microglia; OL, oligodendrocytes; OPC, oligodendrocyte precursor cells; PreOL, pre-myelinating oligodendrocytes; SN, subpallial neurons. Scale bars, 100 μm (**i**,**j** (top)), 10 μm (**i**,**j** (bottom)).

**Extended Data Fig. 2:**
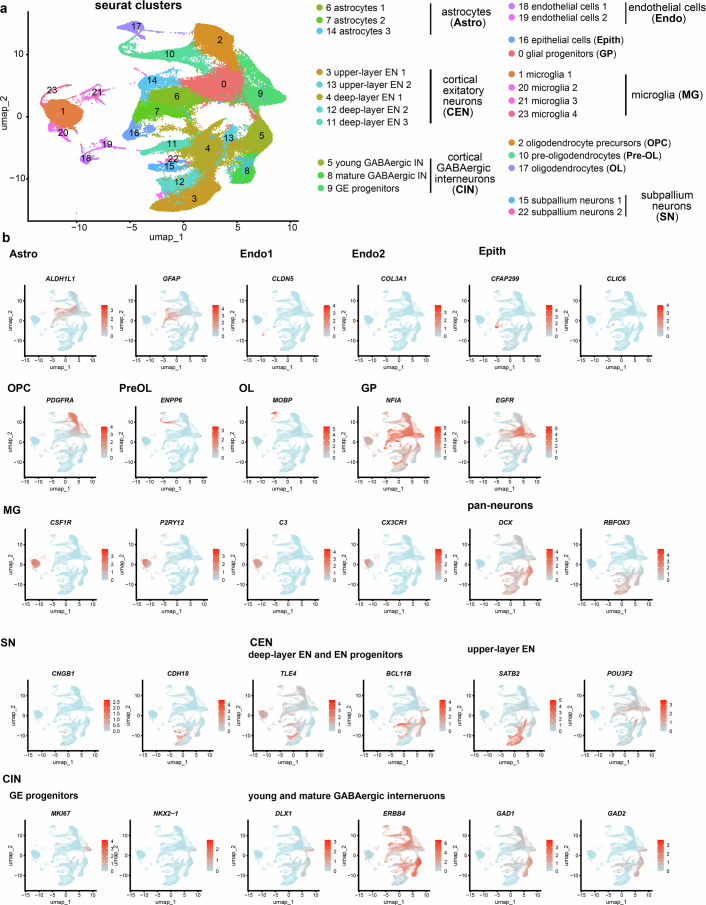
Identification of cell types in human snRNAseq data. **a**, UMAP plots of Seurat clusters and cell type annotations. **b**, Feature plots showing selected canonical markers for different types of cells, including *ALDH1L1* and *GFAP* for astrocytes (Astro), *CLDN5* and *COL3A1* for endothelial cells (Endo), *CFAP299* and *CLIC6* for epithelial cells (Epith), *PDGFRA* for oligodendrocyte precursor cells (OPC), *ENPP6* for pre-myelinating oligodendrocytes (Pre-OL), *MOBP* for oligodendrocytes (OL), *NFIA* and *EGFR* for glia progenitors (GP), *CSF1R*, *P2RY12*, *C3*, and *CX3CR1* for microglia (MG), *DCX* and *RBFOX3* for pan-neurons, *CNGB1* and *CDH18* for subpallium neurons (SN), *TLE4*, *BCL11B*, *SATB2* and *POU3F2* for cortical excitatory neurons (CEN), *MKI67* and *NKX2-1* for GE progenitors, *DLX1*, *ERBB4*, *GAD1*, and *GAD2* for GABAergic interneurons. GE progenitors (cluster 9, *NKX2.1*^+^*MKI67*^+^), young GABAergic interneurons (cluster 5, *DCX*^+^*RBFOX3*^low^*GAD1*^+^ *GAD2*^+^), and mature GABAergic interneurons (*RBFOX3*^+^*DCX*^low^*GAD1*^+^ *GAD2*^+^) were combined as cortical interneurons (CIN) for the following analysis.

To identify the molecular underpinnings of microglial regulation of extended neurogenesis during human interneuron development, we generated a single-nucleus RNA sequencing (snRNA-seq) dataset of the late embryonic stage (GW22–30) to the perinatal stage (postnatal weeks 2–3) using a droplet-based 10X Genomics platform (*n* = 6 donors; Fig. 2a and Supplementary Tables 1 and 2). We focused on human tissues that contained ganglionic eminences, adjacent periventricular regions (containing the Arc, an area of SVZ composed of migratory interneurons^15^) and cortical regions where mature interneurons reside (Fig. 2a and Supplementary Table 1). Neurons were over-represented in snRNA-seq^33^. To better capture both neurons and glia, we adapted flow cytometry-based strategies to enrich microglia through PU.1^+^ and oligodendrocyte lineage cells and MGE progenitors through OLIG2^+^ in addition to DAPI^+^ nuclei^34^ (Fig. 2b and Supplementary Table 1). After quality control and doublet removal (Methods), we recovered 124,411 nuclei with a median of 7,341 unique molecular identifiers (UMIs) and 2,925 genes per cell. We then performed principal component analysis (PCA) of normalized read counts, followed by uniform manifold approximation and projection (UMAP) using Seurat v.5 (ref. ^35^). We identified 11 main cell clusters from our transcriptomic data profiles on the basis of the expression of canonical marker genes (Fig. 2c, Extended Data Fig. 2 and Supplementary Fig. 1). Notably, our results contained 17,342 cells in the microglia cluster and 17,667 cells in the cortical GABAergic interneuron cluster, which includes ganglionic eminence progenitors, young GABAergic interneurons and mature GABAergic interneurons (Extended Data Fig. 2).

**Extended Data Fig. 3:**
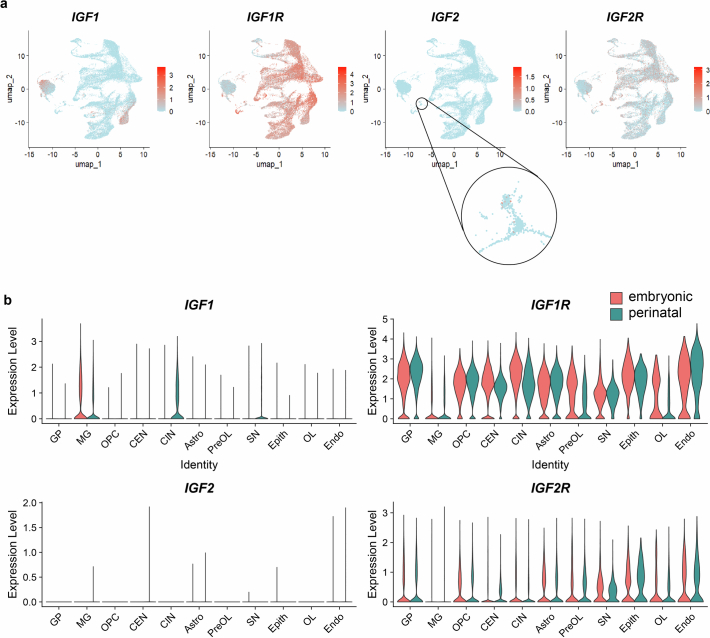
The expression patterns of *IGF1*, *IGF1R*, *IGF2*, and *IGF2R* in developing human samples. **a**, Feature plots showing the expression of *IGF1, IGF1R*, *IGF2*, and *IGF2R* in developing human samples. **b**, Violin plots showing the expression of *IGF1*, *IGF1R*, *IGF2*, and *IGF2R* in embryonic and perinatal human samples.

**Extended Data Fig. 4:**
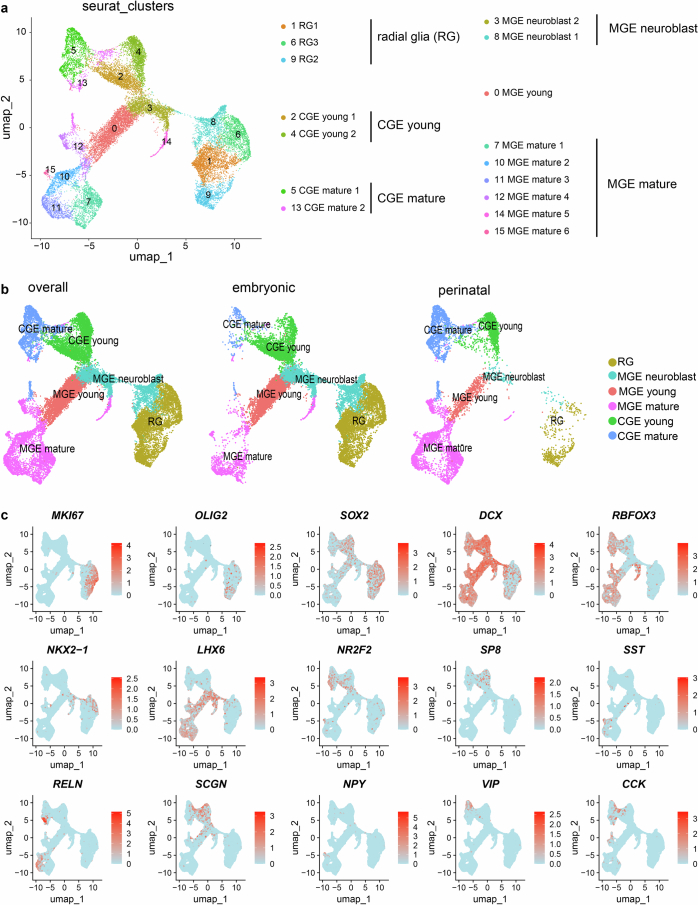
Identification of interneuron subtypes in human snRNAseq data. **a**, Interneuron subtype recognition according to Seurat clustering and canonical markers. **b**, UMAP of interneuron subclusters in the embryonic and perinatal stages. **c**, Feature plots of canonical markers. Radial glia (RG) are cell clusters that express high *MKI67*, *OLIG2*, and *SOX2*; MGE neuroblasts are cell clusters with residual *MKI67*, *SOX2* and *NKX2-1* expression and starting to express *DCX* and *LHX6*, corresponding to the neuroblast cells within DENs in the hMGE. MGE-derived young interneurons (MGE young) are cell clusters that do not express *MKI67* and *NKX2-1*, but express high levels of *DCX* and *LHX6* and low levels of *RBFOX3*. MGE-derived mature interneurons (MGE mature) are cell clusters that express high levels of *RBFOX3* and *LHX6* and low levels of *DCX*. CGE-derived young interneurons (CGE young) are cell clusters that do not express *MKI67*, but express high levels of *DCX*, *NR2F2*, and *SP8*, and low levels of *RBFOX3*. CGE-derived mature interneurons (CGE mature) are cell clusters that express high levels of *RBFOX3*, *NR2F2*, and *LHX6*, and low levels of *DCX*.

To predict cell-type-specific interactions between microglia and other cell types in the developing hMGE, we leveraged a highly curated database of receptor–ligand interactions to predict possible interactions among different types of cells^36^ (Supplementary Fig. 2). This approach also revealed development-related pathways by comparative analysis of cell–cell communications at the late embryonic and perinatal stages (Supplementary Fig. 3). To gain insight into the interaction involved in the microglial regulation of cortical interneurons, we extracted these ligand–receptor pairs with statistical significance (Fig. 2d). Notably, IGF1 and IGF1R constitute the ligand–receptor pair with the highest communication probability, which was predominantly observed during the embryonic stages (Fig. 2d). Chord, violin and feature plots showed that IGF1 was predominantly derived from microglia during the embryonic stage, although it was also generated by interneurons during the perinatal stage (Fig. 2e,f and Extended Data Fig. 3). To clarify which interneuron population expresses IGF1, we conducted interneuron subcluster analysis and identified unique populations, namely radial glia, MGE neuroblasts, MGE-derived young interneurons, MGE-derived mature interneurons, caudal ganglionic eminence (CGE)-derived young interneurons and CGE-derived mature interneurons, on the basis of unbiased clustering and canonical markers (Fig. 2g and Extended Data Fig. 4). We observed that IGF1 was mainly expressed by mature interneurons but was barely detected in interneuron progenitors, including radial glia and MGE neuroblasts (Fig. 2h). These results indicate that microglia are a key source of IGF1 during interneuron proliferation in developing hMGE. Accordingly, IGF1R was highly expressed in interneurons, particularly at the embryonic stage (Fig. 2e,f and Extended Data Fig. 3). Subcluster analysis further confirmed the high expression levels of IGF1R in interneuron progenitors and young interneurons (Fig. 2g,h). IHC showed that in GW22–25 hMGE, IGF1 was specifically expressed in microglia (Fig. 2i), whereas IGF1R was widely expressed in progenitors in the hMGE (Fig. 2j). Taken together, our data indicate that IGF1 is primarily derived from microglia during the embryonic stage and has the potential to support interneuron development in hMGE.

## HPSC-derived MGEOs recapitulate hMGE development

**Fig. 3:**
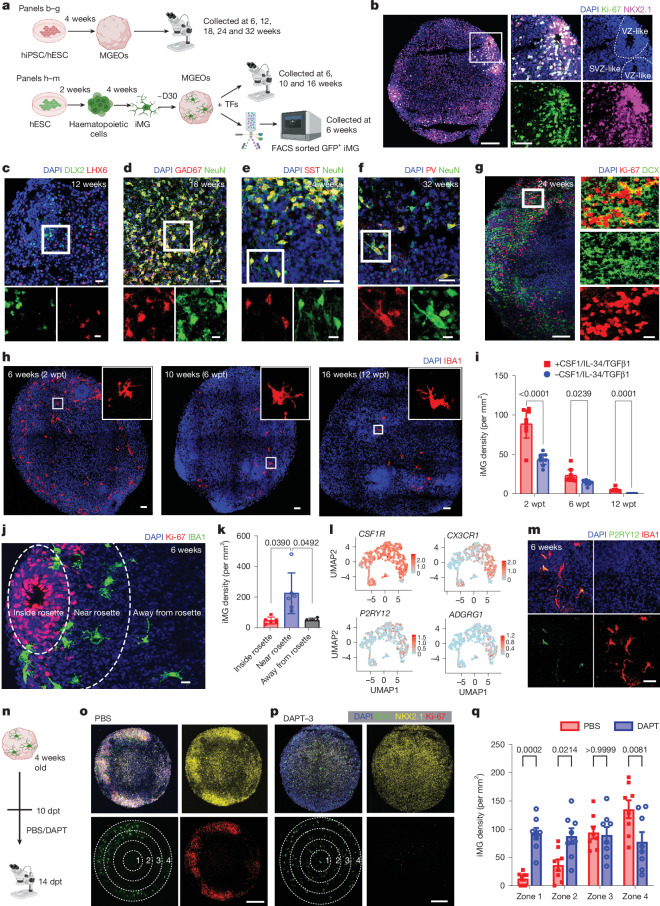
MGEO models hMGE development and interaction between microglia and hMGE progenitors. **a**, Schematic diagram of experimental flow. Transcriptional factors (TFs) include CSF1, IL-34 and TGFβ1. **b**, MGEOs containing ‘rosette’-like proliferating centres, recapitulating the ventricular zone (VZ)-like and SVZ-like areas of hMGE. **c**–**f**, Sequential expression of markers for MGE progenitors and MGE-derived interneurons that mimic the temporal progression of hMGE development in MGEOs including DLX2 and LHX6 (**c**), GAD67 and NeuN (**d**), SST (**e**) and PV (**f**). **g**, Clusters of DCX^+^ neuroblasts sprinkled with Ki-67^+^ cells noted in 24-week-old MGEOs, resembling DEN-like structures in hMGE. **h**,**i**, IHC images (**h**) and bar graphs (**i**) showing iMG survived up to 12 weeks post-iMG transplantation (wpt) in MGEOs. **j**,**k**, IHC images (**j**) and quantification (**k**) showing the distribution of iMG around rosettes. **l**, Feature plots of microglial homeostatic markers expressed in FACS-isolated iMG from 6-week-old MGEOs. **m**, IHC images showing the expression of P2RY12 in iMG. **n**, Schematic outlining the administration of PBS or DAPT from 10 to 14 dpt before the IHC analysis of iMG distribution. **o**–**q**, IHC images of control (**o**) and DAPT-treated MGEOs (**p**) and bar graphs (**q**) showing that DAPT treatment sharply reduced the number of Ki-67^+^ proliferating cells and rosette formation in MGEOs. As proliferation decreased, iMG became more evenly distributed throughout the organoids. The white dashed lines mark the concentric ellipses (zones 1–4) used for binned quantification of iMG density in **q**. For statistics, *n* = 8, 8, 8, 7, 8 and 7 (each group in **i**); *n* = 6 (**k**); and *n* = 8 (**q**). Unpaired two-tailed *t*-test (**i**); one-way repeated measures ANOVA and post hoc Bonferroni’s test (**k**); two-way repeated measures ANOVA and post hoc Bonferroni’s test (**q**); data in **i**,**k** and **q** are shown as mean ± s.e.m. Scale bars, 100 μm (**b** (left),**g** (left)), 50 μm (**b** (middle),**h**,**m**), 25 μm (**c**–**f** (top),**g** (right)), 10 μm (**c**–**f** (bottom)), 20 μm (**j**). Illustrations in **a** and **n** were created using BioRender (https://biorender.com).

**Extended Data Fig. 5:**
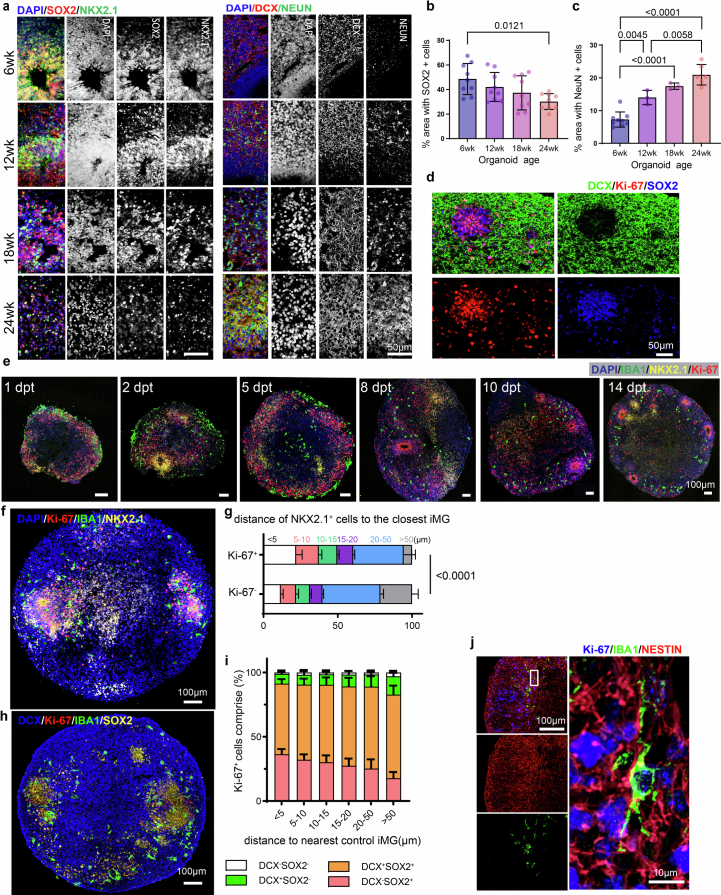
Characterization of MGEOs. **a-c**, MGEOs sequentially express markers for radial glia (SOX2) and postmitotic interneurons (DCX and NeuN) as they mature. SOX2^+^ cells are high at 6 weeks-old and gradually decrease, whereas NeuN^+^ neurons gradually increase as MGEOs age. **d**, Ki-67^+^ cells seen in both SOX^+^DCX^-^ radial glia and SOX2^+^DCX^+^ neuroblasts in 6-week-old MGEOs. **e**, Representative images showing the distribution of iMG in MGEOs at different days post-transplantation. **f**-**g**, IHC images and bar graphs showing iMG distribute closer to NKX2.1^+^Ki-67^+^ proliferating progenitors in comparison to NKX2.1^+^Ki-67^-^ cells in 6-week-old MGEOs. **h-i**, IHC images and bar graphs showing the composition of Ki-67^+^ cells in proximity to iMG in 6-week-old MGEOs. **j**, IHC images showing microglia closely interact with NESTIN^+^ projections in MGEOs. One-way ANOVA and post-hoc Bonferroni’s test in (b) and (c); two-way repeated measures ANOVA for (g) and (i) showed significant interaction effects (P < 0.001 for (i)); N = 4 in (g); N = 5 in (i); data in (b), (c), (g), and (i) were shown as means ± SEM.

To investigate the role of microglia and microglial IGF1 in hMGE development, we generated hPSCs, including both human induced pluripotent stem cells (hiPSCs) and hESC-derived ventral organoids, referred to as MGEOs, adapted from established protocols^37,38^ (Fig. 3a). As expected, our MGEOs had robust NKX2.1^+^ and Ki-67^+^ ventricular zone-like rosettes at 6 weeks of age (Fig. 3b) and demonstrated sequential expression of markers for MGE progenitors and interneurons, including DLX2, SOX2, LHX6, DCX, GAD67, NeuN, SST and PV (Fig. 3c–f and Extended Data Fig. 5a–c), confirming MGE identity and generation of GABAergic interneurons. Notably, Ki-67-expressing cells were distributed in the centre of ventricular zone-like rosettes as well as in the edge and neighbouring SVZ-like regions of rosettes at 6 weeks of age (Fig. 3b and Extended Data Fig. 5d). IHC analysis showed that Ki-67^+^ progenitors comprised both SOX2^+^DCX^−^ radial glia-like cells and SOX2^+^DCX^+^ neuroblasts in 6-week-old MGEOs (Extended Data Fig. 5d), recapitulating the spatial organization of radial glia and proliferating neuroblasts in developing hMGE. We observed clusters of DCX^+^ neuroblasts sprinkled with Ki-67^+^ cells in 24-week-old MGEOs, which may represent DEN-like clusters (Fig. 3g). Taken together, our data indicate that MGEOs can serve as a model to study hMGE development.

Microglia do not appear in situ in organoids. To study the role of microglia in hMGE development, we established microglia-containing MGEOs by transplanting hESC-induced iMG into MGEOs at 4 weeks of age, mimicking the stage when microglia are detected in developing human brains at GW4.5 (refs. ^16,39^) (Fig. 3a). The addition of recombinant human colony-stimulating factor 1 (CSF1), interleukin-34 (IL-34) and transforming growth factor-β1 (TGFβ1) to the culture medium significantly improved iMG survival up to 12 weeks post-transplantation (wpt) (Fig. 3h,i), which is consistent with previous reports^40^. We observed that iMG invaded MGEOs after 24 h, at 1 day post-transplantation (dpt), and reached the organoid centre at 5 dpt. The preferential accumulation around rosette-like proliferative centres became apparent at 8–14 dpt (Extended Data Fig. 5e). We observed that iMG mostly avoided the ventricular zone-like rosettes but accumulated in the SVZ-like neighbouring regions in the MGEOs (Fig. 3j,k). As seen in hMGE in vivo, iMG were in close proximity to Ki-67^+^ and SOX2^+^ progenitors (Extended Data Fig. 5f–i) and intimately interacted with NESTIN^+^ projections (Extended Data Fig. 5j).

**Extended Data Fig. 6:**
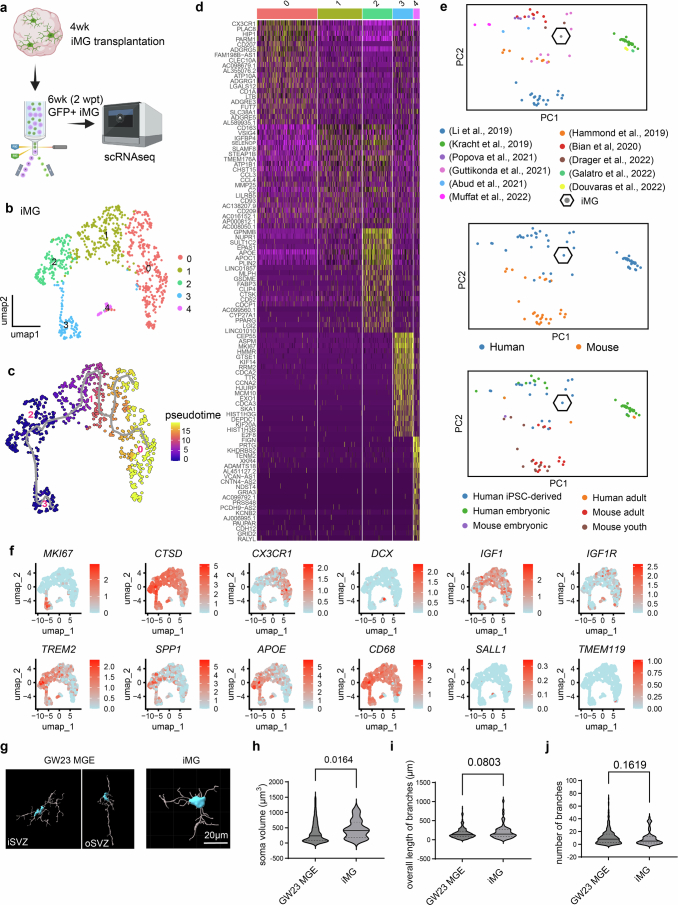
Transcriptomic subcluster analysis reveals that iMG represents distinct states of primary human microglia. **a**, GFP^+^ iMG were isolated from 6-week-old organoids (2 wpt) via FACS and subjected to scRNA-seq. **b**, UMAP visualization of iMG subclusters. **c**, Monocle3-based pseudotime analysis reveals the developmental trajectory of iMG. **d**, Heatmap displaying the top 20 marker genes for each iMG subcluster. **e**, PCA indicates that iMG exhibit transcriptomic profiles similar to published human iPSC-derived and embryonic microglia. **f**, Feature plots of selected marker genes of microglia. Trajectory analysis and marker gene expression suggest that subcluster 0 resembles homeostatic microglia, enriched with *CX3CR1*, *P2RY12*, *CSF1*, and *ADGRG1* (see Fig. 3). Subcluster 2 represents pre-mature and non-homeostatic microglia, marked with *GPNMB*, *CTSD*, *TREM2*, *SPP1*, *APOE*, and *CD68*. Subcluster 3 consists of proliferating microglia enriched in *MKI67*, *RRM2*, and *CDCA2*, while subcluster 4 resembles neuron-associated expressing neuronal genes such as *DCX*, *GRIA3*, and *GRID2*. Notably, *IGF1* expression remains comparable across subclusters, while *SALL1* and *TMEM119* are poorly detected in iMG. **g**, Representative morphologies of primary microglia from GW23 MGE (oSVZ and iSVZ) and iMGs, visualized with IMARIS. **h**, Violin plots showing iMGs exhibited significantly enlarged soma volumes compared to primary microglia from GW23 MGE. Primary microglia from both the oSVZ and iSVZ were included in the analysis. **i**-**j**, Violin plots showing overall lengths (**i**) and numbers (**j**) of branches in primary microglia from GW23 MGE and iMG. iMGs showed comparable levels of ramification with primary microglia from GW23 MGE. Unpaired two-tailed t test; medians (solid line) and quartiles (dash line) were shown in the violin plots (h)-(j). Illustration in **a** was created using BioRender (https://biorender.com).

To further document the properties of the transplanted iMG, we performed single-cell RNA sequencing (scRNA-seq) analysis of iMG and found that they demonstrated similar developmental trajectory and heterogeneity as primary embryonic human microglia^39,41–50^ (Extended Data Fig. 6a–e). They expressed homeostatic microglial markers, such as *CSF1R*, *CX3CR1*, *P2RY12*, as well as *ADGRG1* (Fig. 3l,m), one of the few genes that define yolk sac-derived ‘true’ microglia^51^. Notably, we detected little, if any, *SALL1* and *TEME119* transcripts in iMG (Extended Data Fig. 6f), which is consistent with previous studies^40,52^. Morphologically, the transplanted iMG demonstrated a larger soma volume but a similar ramification as human primary microglia in hMGE around GW23 (Extended Data Fig. 6g–j).

It is intriguing why microglia exhibited a preferential distribution in the SVZ of hMGE and the proliferating zone of MGEOs. Human microglia have low proliferation capacity at this developmental stage^53^. Our snRNA-seq results showed that 1.64% (75 of 4,561) and 0.15% (14 of 9,631) of microglia were Ki-67^+^ at GW23–30 and postnatal weeks 2–3, respectively. We did not observe any Ki-67^+^ microglia in the GW23 MGE in our immunostaining (*n* = 3). These results challenged proliferation as the primary mechanism for microglial accumulation in SVZ. To probe whether proliferating progenitors promote microglial chemotaxis towards the proliferating zone, we blocked cell proliferation by administering the Notch pathway inhibitor DAPT^54^ from 10 to 14 dpt (Fig. 3n). The DAPT treatment effectively eliminated cell proliferation and rosette formation (Fig. 3o,p). We observed that iMG was evenly distributed in organoids treated with DAPT (Fig. 3p,q). Taken together, our results indicate that microglia are likely to migrate to the SVZ of hMGE and the proliferating zone of MGEOs in response to proliferating progenitors.

## Microglia promote MGE neurogenesis

**Fig. 4:**
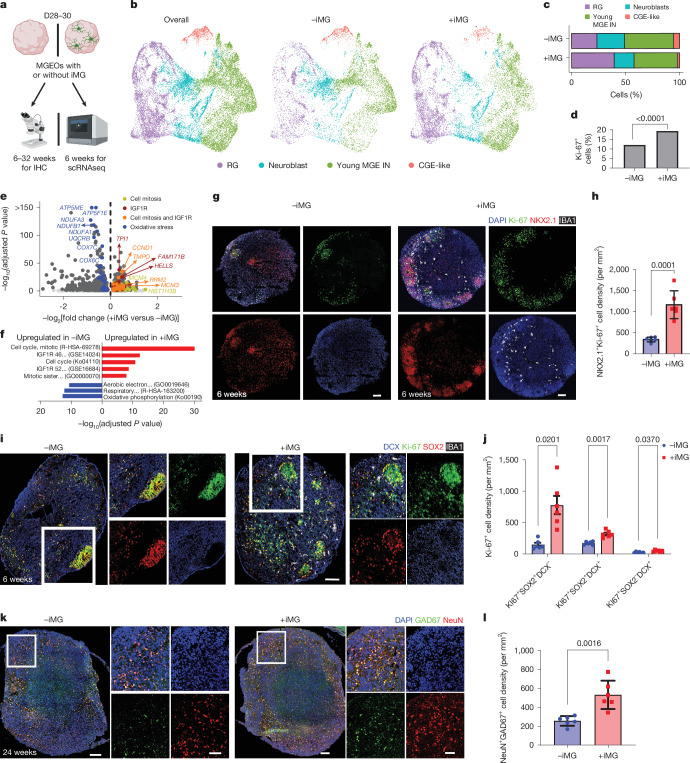
Microglia promote MGE proliferation and interneuron production. **a**, Experimental diagrams to examine iMG effects in MGEOs. **b**, UMAP plots showing cell type clusters from 6-week-old MGEOs with and without iMG. **c**, The proportion of RG significantly increased in MGEOs transplanted with iMG (*P* < 0.0001). **d**, The percentage of Ki-67^+^ nuclei significantly increased in MGEOs with iMG. **e**, Volcano plot showing DEGs in the RG of MGEOs with and without iMG. **f**, Upregulated DEGs in MGEOs with iMG are enriched in pathways and gene ontologies related to cell mitosis and the IGF1R signalling pathway; upregulated DEGs in MGEOs without iMG were enriched in pathways and gene ontologies related to oxidative stress. See Methods for the full names of pathways. **g**,**h**, IHC images (**g**) and bar graph (**h**) showing that iMG increased the density of NKX2.1^+^Ki-67^+^ progenitors in 6-week-old MGEOs. **i**,**j**, IHC images (**i**) and bar graph (**j**) showing iMG increased the density of SOX2^+^DCX^−^ radial glia in 6-week-old MGEOs. **k**,**l**, IHC images (**k**) and bar graph (**l**) showing that iMG increased the density of NeuN^+^GAD67^+^ interneurons in 18-week to 24-week-old MGEOs. For statistics, *n* = 6 (**h**); *n* = 6 (**j**); *n* = 6 (**l**); *χ*^2^ test (two-sided), 2,221 (RG cell number) of 9,456 (overall cell number) versus 4,605 of 11,680, *χ*^2^ = 607.1, *P* < 0.0001 (**c**); *χ*^2^ test (two-sided), 1,146 (Ki-67^+^ cell number) of 9,456 (overall cell number) versus 2,269 of 11,680, *χ*^2^ = 206.0 (**d**); *χ*^2^ tests in **c** and **d** were on the basis of the fractions of targeted cells among the total cells recovered in scRNA-seq data from 6-week-old organoids. The adjusted *P* value in **e** was calculated on the basis of the Seurat-default non-parametric Wilcoxon rank-sum test. The adjusted *P* value in **f** was calculated in Enrichr, using Benjamini–Hochberg correction. Unpaired two-tailed *t*-test in **h** and **l**. Two-way repeated measures ANOVA and post hoc Bonferroni’s test in **j**. Data in **h**,**j** and **l** are shown as mean ± s.e.m. RG, radial glia; IN, interneurons. Scale bars, 100 μm (**g**,**i**,**k** (main image)), 50 μm (**k** (zoomed image)). Illustration in **a** was created using BioRender (https://biorender.com).

**Extended Data Fig. 7:**
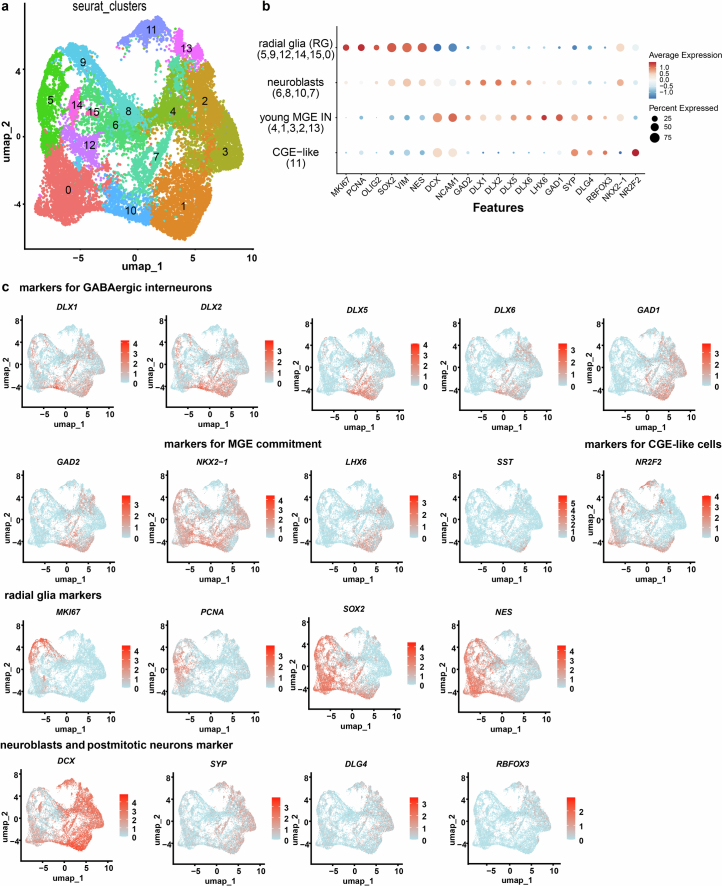
Identification of cell types in 6-week-old MGEOs. **a**, UMAP plots of unbiased clustering of MGEOs. **b**, Dot plots showing the level and cell percentage of canonical marker expression. **c**, Feature plots showing the expression of canonical markers. Radial glia (RG) are recognized as cell clusters that express high *MKI67*, *PCNA*, *OLIG2*, *SOX2*, *VIM*, and NESTIN (*NES*); neuroblasts are recognized as transiting clusters expressing both progenitor markers such as *MKI67*, *PCNA*, *SOX2*, and *NES*, as well as the young postmitotic neuron marker *DCX*. Young MGE-derived GABAergic interneurons (Young MGE IN) are recognized as clusters that express *DCX*, *NCAM*, *GAD1*, *GAD2*, *DLX1*, *DLX2*, *DLX5*, *DLX6*, and low levels of *SYP*, *DLG4*, and *RBFOX3*. Notably, without a FACS-based enrichment strategy, iMG were barely detected in this scRNA-seq result, likely due to their low abundance.

**Extended Data Fig. 8:**
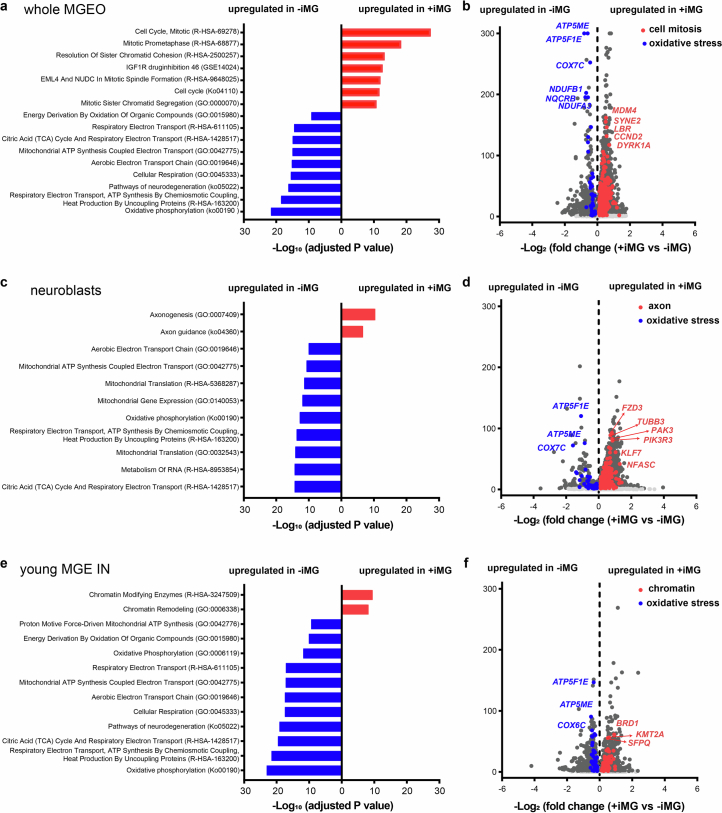
DEGs and pathway analysis. **a**-**b**, Pathway enrichment analysis plot (a) and volcano plot (b) of whole MGEO showing a higher expression of genes related to cell mitosis and lower expression of genes related to oxidative stress in MGEOs with iMG. **c**-**d**, Pathway enrichment analysis plot (c) and volcano plot (d) of neuroblasts cluster revealing an increased expression of genes related to axon development and decreased expression of genes related to oxidative stress in MGEOs with iMG. **e**-**f**, Pathway enrichment analysis plot (e) and volcano plot (f) of young MGE-derived GABAergic interneuron (young MGE IN) populations showing the presence of iMG leads to an increased the expression of genes related to chromatin metabolism and decreased expression of genes related to oxidative stress. The adjusted P value in (a), (c), and (e) was calculated in Enrichr, using Benjamini-Hochberg correction. The adjusted P value in (b), (d), and (f) was conducted based on the Seurat-default non-parametric Wilcoxon rank sum test.

To investigate microglial regulation of interneuron development, we conducted scRNA-seq of 6-week-old MGEOs with and without transplanted iMG at 4 weeks of age (Fig. 4a). We recovered 21,136 cells, with a median of 5,045 UMIs and 2,629 genes per cell. On the basis of unbiased clustering and canonical marker genes, we identified clusters of radial glia, neuroblasts, young MGE-derived GABAergic interneurons and a small group of CGE-like cells (Fig. 4b and Extended Data Fig. 7). Notably, without a fluorescence-activated cell sorting (FACS)-based enrichment strategy, iMG were barely detected in this scRNA-seq result, probably because of their low abundance. We observed a significant increase in the proportion of radial glia and a significantly higher percentage of Ki-67^+^ cells in MGEOs transplanted with iMG (Fig. 4c,d), indicating the role of iMG in promoting progenitor proliferation. Differentially expressed gene (DEG) analysis showed that upregulated genes in MGEOs transplanted with iMG were enriched in pathways and gene ontologies related to cell mitosis (Fig. 4e,f and Extended Data Fig. 8). The downregulated genes in MGEOs with iMG were largely enriched in pathways and gene ontologies related to oxidative stress (Fig. 4e,f and Extended Data Fig. 8), as shown in previous studies^45,55^. Additionally, upregulated genes in radial glia were significantly enriched in Gene Expression Omnibus (GEO) terms related to IGF1R downstream signalling (Fig. 4e,f), such as *CCND1*, *TMPO*, *RRM2* and *MCM3*, in MGEOs with iMG, suggesting a role for IGF1–IG1R in microglial regulation of interneuron progenitor proliferation.

**Extended Data Fig. 9:**
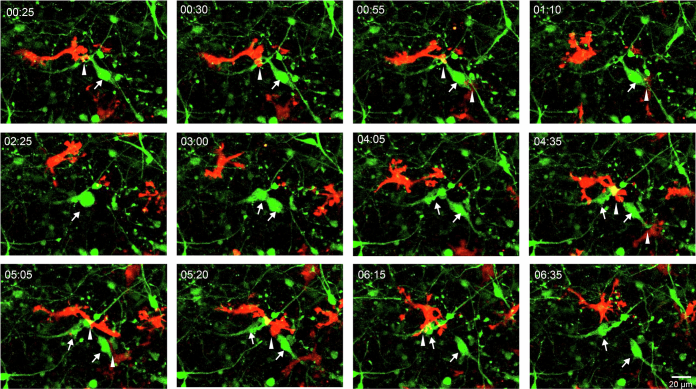
Time-lapse live imaging reveals active interactions between iMG and proliferating progenitors. Arrows indicate cell proliferation, while white triangles highlight interactions between iMG (red, tdtomato labelled) and proliferating mother and daughter cells (green, NKX2.1-GFP). The recording time is shown in the top left corner of each image, formatted as ‘hour:minutes’.

**Extended Data Fig. 10:**
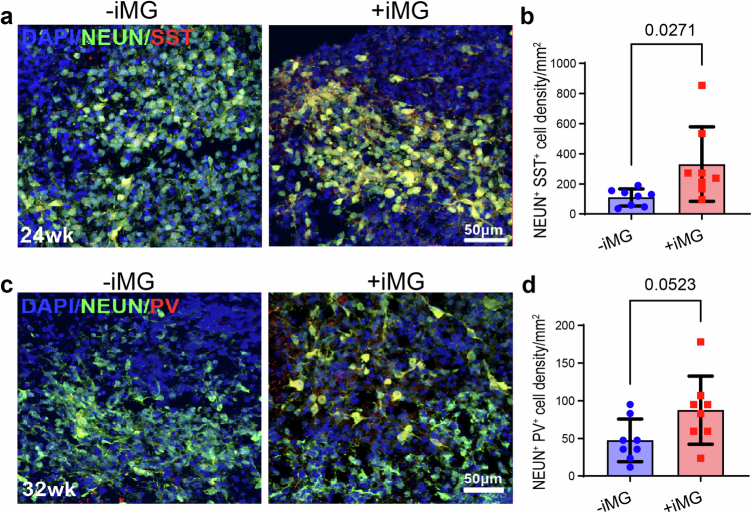
iMG promote MGE-derived interneuron production. **a**-**b**, Representative IHC images and bar graphs showing that the presence of iMG results in higher density of SST^+^NeuN^+^ interneurons in 24-week-old MGEOs. **c**-**d**, Representative IHC images and bar graphs showing iMG transplantation leads to a trend in higher density of PV^+^NeuN^+^ interneurons in 32-week-old MGEOs. N = 8 in (b) and (d); unpaired two-tailed t-test in (b) and (d); data in (b) and (d) were shown as means ± SEM.

We next conducted IHC to characterize the effect of iMG on interneuron development in MGEOs. Using Ki-67 and NKX2.1 as markers, we observed that the density of proliferating MGE progenitors was significantly increased in 6-week-old MGEOs transplanted with iMG (Fig. 4g,h and Supplementary Fig. 4). Further analysis revealed that the presence of iMG significantly increased the density of proliferating radial glia (Ki-67^+^SOX2^+^DCX^−^) and neuroblasts (Ki-67^+^SOX2^+^DCX^+^) (Fig. 4i,j). To document the dynamic interaction between iMG and proliferating progenitors, we generated MGEOs using NKX2.1-GFP hESCs^56^ and transplanted them with tdTomato-labelled iMG. We observed active interactions between microglia and NKX2.1^+^ cells. We captured one dividing NKX2.1-GFP cell after direct contact from microglia (Extended Data Fig. 9 and Supplementary Video 1). We found that iMG transplantation significantly increased the density of NeuN^+^GAD67^+^ mature interneurons in 18-week- to 24-week-old organoids (Fig. 4k,l), as well as increased NeuN^+^SST^+^ and a trend of increased NeuN^+^PV^+^ MGE-derived subtypes of interneurons in 24-week and 32-week-old organoids (Extended Data Fig. 10). Taken together, our data indicate that microglia promote the proliferation and generation of MGE-derived interneurons.

## Microglial IGF1 promotes MGE proliferation

**Fig. 5:**
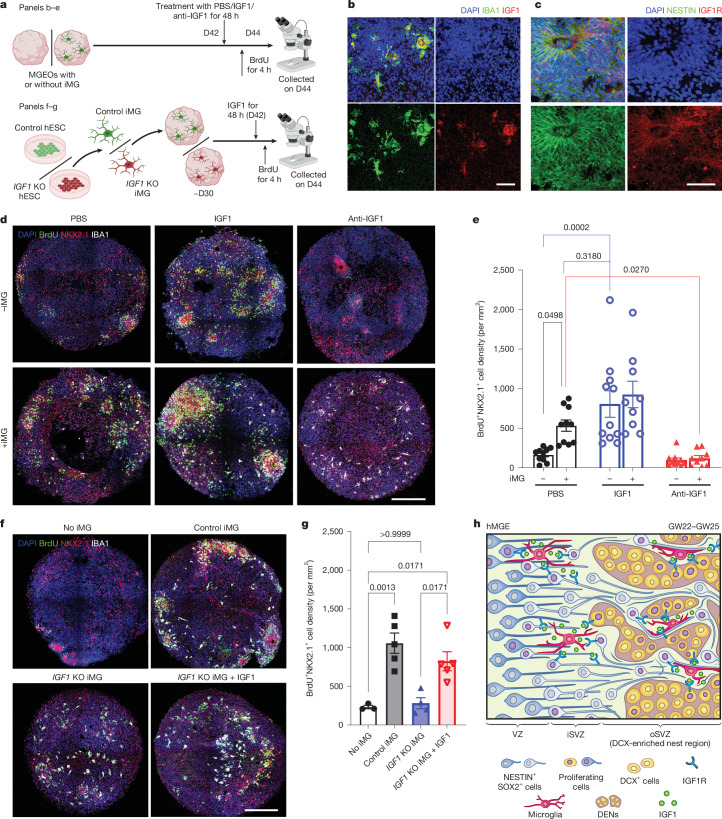
Microglia-derived IGF1 promotes MGE proliferation in MGEOs. **a**, Experimental schematics. **b**, IHC images indicating the specific expression of IGF1 in iMG of 6-week-old MGEOs. **c**, IHC images showing the expression of IGF1R in progenitors in the rosettes of 6-week-old MGEOs. **d**,**e**, IHC images (**d**) and bar graphs (**e**) showing that IGF1 promotes MGE progenitor proliferation in MGEOs without iMG to the level seen in MGEOs with iMG, and that IGF1-neutralizing antibody treatment abolished iMG-mediated progenitor proliferation. **f**,**g**, IHC images (**f**) and bar graphs (**g**) showing the inability of *IGF1* knockout (KO) iMG to promote MGEO progenitor proliferation, which can be rescued by the addition of recombinant IGF1 treatment. **h**, Graphical abstract showing microglial regulation of interneuron progenitor proliferation through IGF1 in hMGE. For statistics, *n* = 10, 11 and 9 for MGEO −iMG; *n* = 10, 9 and 9 for MGEO +iMG (**e**). *n* = 3, 5, 5 and 5 (**g**). Two-way ANOVA (**e**), one-way ANOVA (**g**) and post hoc Bonferroni’s test for selected comparisons. Data in **e** and **g** are shown as mean ± s.e.m. Scale bars, 50 μm (**b**,**c**), 200 μm (**d**,**f**). Illustration in **a** was created using BioRender (https://biorender.com).

We next investigated whether microglia exert their function in hMGE development through IGF1 using our neuroimmune MGEOs (Fig. 5a). We showed that IGF1 was specifically detected in iMG, whereas IGF1R was highly expressed in NESTIN^+^ radial glia in 6-week-old MGEOs (Fig. 5b,c), as observed in hMGE (Fig. 2i,j).

**Extended Data Fig. 11:**
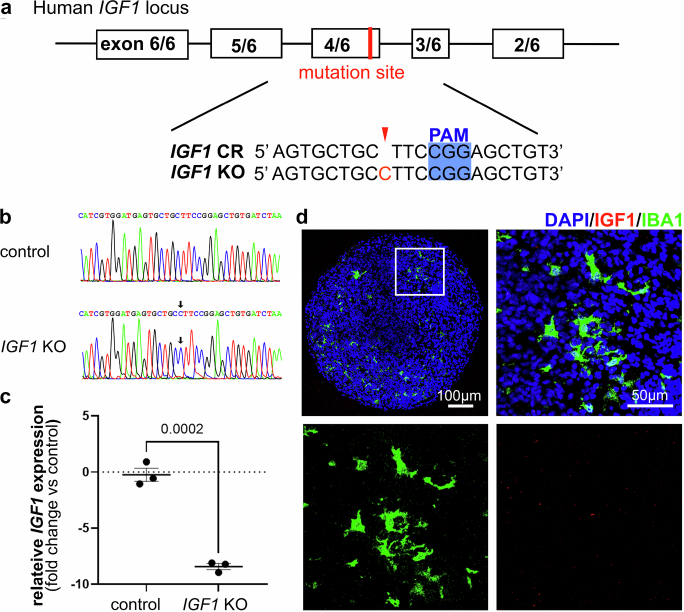
Establishment of *IGF1* loss-of-function mutation stem cell line. **a**, Strategy for generation of *IGF1* loss-of-funciton mutation cell line with Cripsr/Cas9-based NHEJ. **b**, Sanger sequencing results confirming *IGF1* mutation. **c**, RT-qPCR of *IGF1* expression in iMG. **d**, IHC of iMG in 6-week-old organoids showing no IGF1 is detected in iMG derived from *IGF1* KO cell line. N = 3 (three independent prepartions of iMG) in (c), unpaird two-tailed t-test. Data in (c) were shown as means ± SEM.

**Extended Data Fig. 12:**
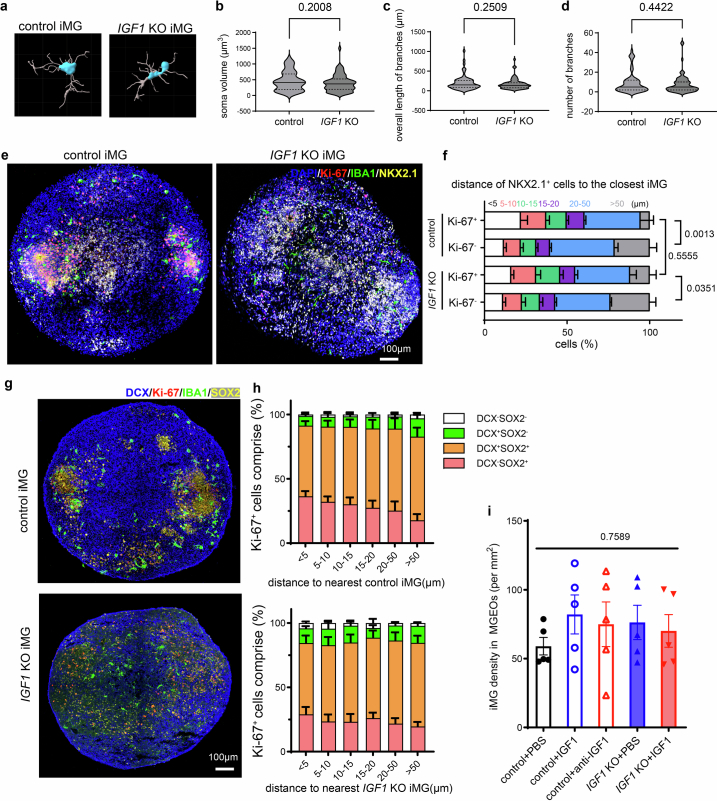
*IGF1* KO has minimal impact on iMG morphology, distribution and density. **a**, Representative control and *IGF1* KO microglial morphology visualized with IMARIS. **b-d**, Violin plots showing *IGF1* KO does not significantly alter soma volume (b), total branch length (c), or branch number (d) in iMG. **e-f**, IHC images and bar graphs showing that *IGF1* KO iMGs, like control iMGs, localize closer to NKX2.1^+^Ki-67^+^ proliferating progenitors than to NKX2.1^+^Ki-67^-^ cells in MGEOs. **g-h**, IHC images and bar graphs showing a high percentage of SOX2^+^ cells among Ki-67^+^ proliferating progenitors located near *IGF1* KO iMGs, similar to control iMGs. **i**, iMG density in 6-week-old MGEOs is not significantly different among different IGF1 treatment and iMG genotyping conditions. Unpaired two-tailed t-test in (b)-(d); P value of interaction effect of distance x Ki-67 were labelled, two-way repeated measures ANOVA in (f); N = 4,5 in (h); N = 5 in (i), one-way ANOVA. Data in (b)-(d) were shown as violin plots with medians (solid line) and quartiles (dash line) indicated. Data in (f), (h), (i) were shown as means ± SEM.

**Extended Data Fig. 13:**
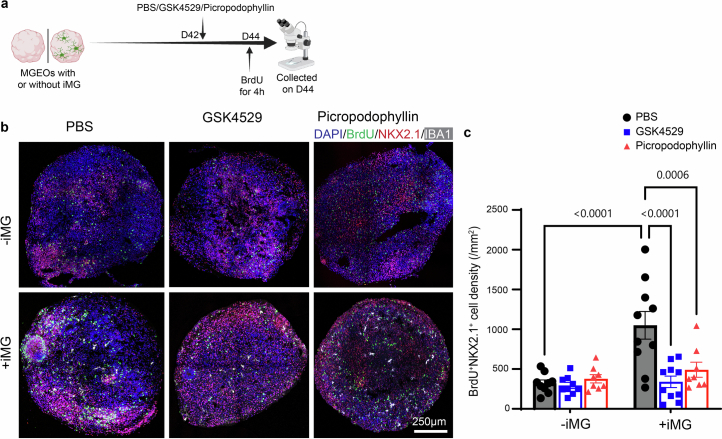
IGF1R inhibition prevents iMG-induced promotion of MGE proliferation. **a**, Experimental schematic diagram showing that MGEOs transplanted with or without iMG were treated with PBS or IGF1R inhibitors -GSK4529 and Picropodophyllin- for two days at week 6. BrdU was administered during the last four hours to label proliferating cells. **b-c**, IHC images and bar graphs showing that GSK4529 and Picropodophyllin prevented iMG-induced promotion of MGE proliferation. N = 9, 9, 8; 10,10, 8; two-way ANOVA and post-hoc Bonferroni’s test for selected comparisons. Data in (c) were shown as means ± SEM. Illustration in **a** was created using BioRender (https://biorender.com).

To examine the role of IGF1 in hMGE development, we treated 6-week-old MGEOs, transplanted with or without iMG at 4 weeks of age, with either a carrier control (phosphate-buffered saline (PBS)), recombinant human IGF1 proteins (IGF1) or IGF1-neutralizing antibodies (anti-IGF1) for 48 h. BrdU was added during the last 4 h of the 48-h treatment for precise evaluation of cell proliferation cells (Fig. 5a). We observed that the presence of iMG increased the density of BrdU^+^NKX2.1^+^ cells in MGEOs (Fig. 5d,e). IGF1 treatment drastically increased the density of BrdU^+^NKX2.1^+^ cells in MGEOs without iMG transplantation, whereas IGF1-neutralizing antibodies abolished the elevated density of BrdU^+^NKX2.1^+^ cells in organoids with iMG (Fig. 5d,e). To definitively demonstrate that IGF1 is the key regulator mediating microglial regulation of interneuron development, we generated *IGF1* loss-of-function mutation (*IGF1* knockout) hESC lines using CRISPR–Cas9-based non-homology end joining (Extended Data Fig. 11). We then generated MGEOs transplanted with no iMG, control iMG or *IGF1* knockout iMG at 4 weeks of age and examined MGE progenitor proliferation by quantifying the density of BrdU and NKX2.1 double-positive cells at 6 weeks of age (Fig. 5a). We observed that MGE progenitor proliferation increased in MGEOs transplanted with control iMG but not in those transplanted with *IGF1* knockout iMG (Fig. 5f,g). The addition of recombinant human IGF1 rescued the phenotype associated with *IGF1* knockout iMG (Fig. 5f,g). On the other hand, we did not observe any significant changes in the morphology, distribution or density of iMG in MGEOs following *IGF1* knockout (Fig. 5f and Extended Data Fig. 12). To investigate IGF1 signalling through IGF1R, we administered IGF1R inhibitors (GSK4529 (ref. ^57^) and picropodophyllin^58,59^) to MGEO cultures. We observed that both inhibitors abolished iMG-induced MGE proliferation (Extended Data Fig. 13). In summary, our results indicate that iMG exert their function in promoting MGE progenitor proliferation through the IGF1–IGF1R pathway.

**Extended Data Fig. 14:**
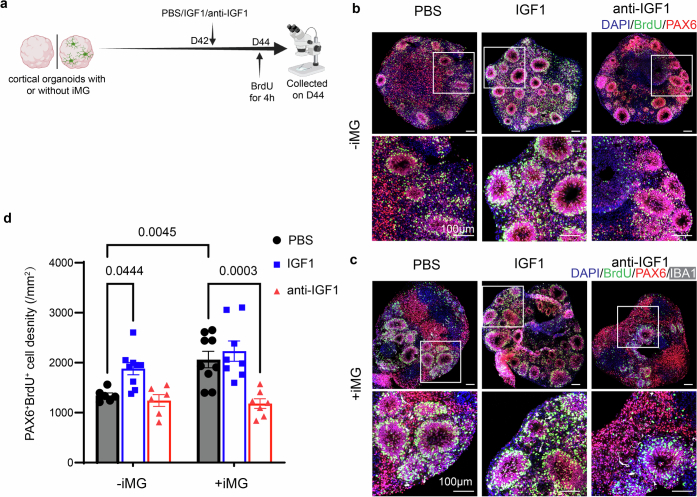
iMG-derived IGF1 promotes PAX6^+^ progenitor proliferation in cortical organoids. **a**, Experimental schematic diagram showing cortical organoids transplanted with or without iMG were treated with PBS, IGF1, and anti-IGF1 neutralizing antibodies for two days at week 6. BrdU was administered during the last four hours to label proliferating cells. **b**, IHC images showing BrdU^+^PAX6^+^ proliferating progenitors in cortical organoids without iMG. **c**, IHC images showing BrdU^+^PAX6^+^ proliferating progenitors in cortical organoids with iMG. **d**, Bar graphs showing that iMG and IGF1 significantly increased the density of PAX6^+^BrdU^+^ proliferating progenitors in cortical organoids. IGF1-neutralizing antibody treatment abolished iMG-mediated progenitor proliferation. N = 6, 9, 6; 9, 8, 7; two-way ANOVA and post-hoc Bonferroni’s test for selected comparisons. Data in (d) were shown as means ± SEM. Illustration in **a** was created using BioRender (https://biorender.com).

To examine whether IGF1 can promote cortical radial glial proliferation and projection neurogenesis, we generated cortical neuroimmune organoids and treated 6-week-old organoids with PBS, IGF1 or anti-IGF1. The iMG transplantation and IGF1 treatment significantly increased the density of PAX6^+^BrdU^+^ cells, whereas anti-IGF1 significantly reduced it, indicating that iMG and iMG-derived IGF1 enhanced PAX6^+^ neural progenitor proliferation in cortical organoids (Extended Data Fig. 14).

**Extended Data Fig. 15:**
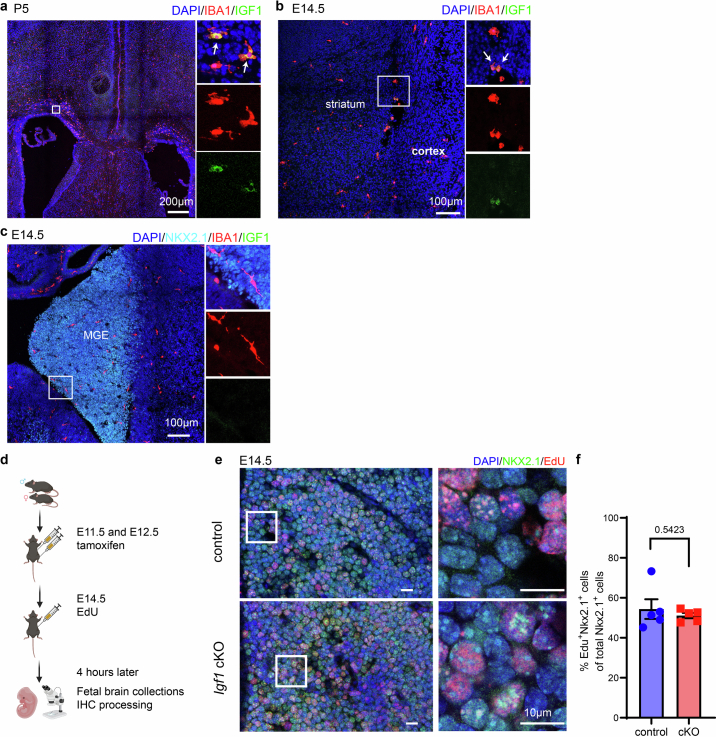
Microglial IGF1 is not involved in MGE proliferation in mice. **a**, IGF1 is expressed by ATM located at developing white matter at P5. **b**, IGF1 is expressed by ATM-like microglia located at cortico-striato-amygdalar boundary at E14.5. Arrows indicate IGF1^+^ microglia in **a** and **b**. **c**, IGF1 is not detectable in microglia located in the mouse MGE at E14.5. **d**, Pregnant dams received IP tamoxifen injections at E11.5 and E12.5, followed by an EdU injection at E14.5, four hours prior to fetal brain collection and subsequent IHC processing. **e-f**, IHC images and bar graph showing that microglial *Igf1* cKO mice (*Igf1*^*f/f*^*, Cx3cr1*^*CreERt/+*^) show a comparable MGE proliferation to their littermate controls. N = 5 and 5 from three litters. Unpaired two-tailed t-test. Data in (f) were shown as means ± SEM. Illustration in **d** was created using BioRender (https://biorender.com). Source data

Finally, we probed whether the microglial IGF1 mechanism of the supporting interneuron development is species-specific. We first examined the expression of IGF1 protein in mouse microglia. Consistent with the literature, we found that IGF1 is expressed by axon tract-associated microglia (ATM)^42^ in the developing white matter at P5 (Extended Data Fig. 15a) and by ATM-like microglia^60^ at the cortico-striato-amygdalar boundary on embryonic day 14.5 (Extended Data Fig. 15b). However, we did not detect IGF1 protein in microglia located in the mouse MGE on embryonic day 14.5 (Extended Data Fig. 15c). Previous cross-species single-cell studies revealed that IGF1 is expressed at significantly higher levels in human microglia than in mice and other rodents^61^. Next, we investigated MGE proliferation in microglial *Igf1* conditional knockout (cKO) mice (*Igf1*^*f*/*f*^, *Cx3cr1*^*CreERt*/+^). We injected tamoxifen on embryonic days 11.5 and 12.5 to induce microglial *Igf1* knockout, followed by EdU pulse labelling of proliferating progenitors 4 h before brain collection on embryonic day 14.5, the peak time for late MGE neurogenesis^62^. Microglial *Igf1* cKO did not affect MGE proliferation on embryonic day 14.5 (Extended Data Fig. 15d–f). Combined with the differential distribution of microglia and proliferating progenitors in the MGE between humans and mice (Fig. 1 and Extended Data Fig. 1), our results indicate a species-specific mechanism for microglial regulation of MGE proliferation.

## Discussion

In summary, our findings provide critical insights into the role of microglia in the development of hMGE and their influence on GABAergic interneurons (Fig. 5h). We demonstrated that microglia exhibit a species-specific distribution pattern, closely interacting with proliferating progenitors in hMGE (Fig. 1). Further studies on the basis of transcriptomic analysis (Fig. 2) and 3D MGEO models demonstrated a clear function of microglia in hMGE progenitor proliferation through the IGF1 pathway (Fig. 5). By contrast, in mice, microglia are more abundant in the ventricular zone of MGE (Extended Data Fig. 1a–f), have minimal IGF1 expression (Extended Data Fig. 15c) and display independence of IGF1 in MGE progenitor proliferation (Extended Data Fig. 15d–f). These findings indicate an evolutionary adaptation of microglial function to support the increased demand for interneurons in the human cortex.

This study uncovered a developmentally specific role of microglia in IGF1-mediated neurogenesis in hMGE. Although the neurotrophic role of IGF1 has been reported^63–72^, we found that microglia serve as a key source of IGF1 during early hMGE development. IGF1 expression in microglia decreased from the embryonic phase to the postnatal period (Fig. 2f), which is consistent with the literature^73^. As microglial IGF1 expression declines with age, IGF1 is expressed by mature interneurons (Fig. 2h), indicating a developmental switch in its cellular source. The role of microglia in cortical neurogenesis, particularly in humans, remains unclear. Although microglia have been shown to promote embryonic cortical neurogenesis in mice^74–76^ and hPSC-derived cortical organoids (Extended Data Fig. 14), their contribution to human cortical development in vivo is unclear. Compared with rodents, microglial distribution varies significantly across cortical regions in macaques^77^ and has not been consistently reported in the human cortex^53,77^. These differences highlight the need for further studies to clarify the extent and function of microglia in human cortical neurogenesis.

As a close family member of IGF1, IGF2 has been implicated in neurogenesis in mice^78,79^. Its source is largely endothelial cells^78,79^. Our snRNA-seq analysis showed that in humans, IGF2 is expressed at low levels and is specifically localized to endothelial cells (Extended Data Fig. 3), mirroring the pattern observed in mice^78,79^. IGF2 receptors (IGF2R) are expressed in interneuron progenitors (Extended Data Fig. 3), suggesting that IGF2 signalling may also contribute to developmental neurogenesis, although not through the microglia–progenitor axis.

Studies in rodents have shown a region-specific interaction between the radial glia and microglia. For example, microglia wrap their processes around radial glial projections in the embryonic mouse hypothalamus and respond to immune challenges^80^. Radial glial endfeet contact meningeal microglial precursors and regulate microglial development through integrin αVβ8–TGFβ1 signalling in the embryonic mouse cortex^81^. Our findings indicate that microglia closely interact with radial glia in the SVZ of hMGE (Fig. 1l). Furthermore, our data from neuroimmune organoid models indicate that microglia enhance the proliferation of radial glia by secreting IGF1 (Figs. 4 and 5), highlighting a vital neuroimmune mechanism in brain development. Further studies are needed to understand the mechanism underlying the intimate and preferential association between radial glia and microglia in hMGE.

Brain organoids have been used to model various aspects of brain development and disease in vitro^52,82–88^. This in vitro model enables mechanistic studies of human-specific aspects of brain development. We adapted a ventral telencephalon-oriented organoid model and demonstrated the sequential presence of MGE progenitors, hMGE unique DEN-like organization and MGE-derived PV^+^ and SST^+^ interneurons, resembling the developmental trajectory of the developing hMGE (Fig. 3b–g). The limitations of brain organoid models include the lack of endogenous microglia and short survival duration of transplanted microglia. Here we showed that the presence of trophic factors, recombinant human CSF1, IL-34 and TGFβ, supports iMG survival up to 12 wpt. These iMG resemble human primary microglial morphology, transcriptomics and physical proximity to proliferating radial glia and progenitors (Fig. 3j–m and Extended Data Figs. 5f–j and 6). Thus, our newly established neuroimmune MGEOs serve as a reliable model for studying the microglial regulation of hMGE development.

The results of this study have significant implications for understanding the development of GABAergic interneurons in the human brain and the potential aetiology of neurological and psychiatric disorders associated with microglial and interneuron dysfunction. Epidemiological studies have shown lower levels of IGF1 in the cerebrospinal fluid of children with autism^89,90^. Further human and mouse studies have demonstrated the potential efficacy of IGF1 administration in ameliorating autistic features^91–95^. This study indicates that microglia are the main sources of IGF1 in the embryonic human brain (Fig. 2e–i and Extended Data Fig. 3), and that IGF1 promotes interneuron production (Figs. 4 and 5), suggesting the possibility that low levels of IGF1 could result in interneuron deficits.

## Limitations of the study

Owing to the limitations of current technologies, we were unable to clarify the real-time dynamics of microglial migration into hMGE proliferative zones, their live interactions with progenitor cells and their direct effects on progenitor division.

## Methods

### Human tissue samples

De-identified human specimens were collected from the Autopsy Service in the Department of Pathology at the University of California San Francisco (UCSF) (Supplementary Table 2) with previous patient consent in strict observance of the legal and institutional ethical regulations. Autopsy consents and all protocols for human prenatal brain tissue procurement were approved by the Human Gamete, Embryo and Stem Cell Research Committee (Institutional Review Board GESCR no. 10-02693) at UCSF. All specimens received diagnostic evaluations by a board-certified neuropathologist as control samples and were free of brain-related diseases. The diagnostic panel included assessments of neural progenitor and immune cells using IHC to ensure that all control cases were not affected by any inflammatory diseases. Tissues used for snRNA-seq were snap-frozen, either on a cold plate placed on a slab of dry ice or in isopentane on dry ice. Tissues later used for IHC were cut coronally into 1-mm tissue blocks, fixed with 4% paraformaldehyde (PFA) for 2 days, cryoprotected in a 15–30% sucrose gradient, embedded in optimal cutting temperature (OCT; SciGen; 4586) compound, sectioned at 30 μm using a Leica cryostat and mounted onto glass slides.

### Authentication of cell lines used

All hiPSC and hESC lines used in this study were karyotyped and regularly tested for *Mycoplasma*. The eWT-1323-4 hiPSC line^84^ (female; Research Resource Identifier (RRID): CVCL_0G84) was obtained from the Conklin Laboratory (UCSF). WA09/H9 (female; RRID: CVCL_9773; National Institutes of Health (NIH) registration number: NIHhESC-10_0062) and WA01/H1 (male; RRID: CVCL_9771; NIH registration number: NIHhESC-10-0043) were obtained from the WiCell Research Institute. NKX2.1-GFP hESC line^56^ (female) was obtained from Murdoch Children’s Research Institute and Monash University.

### Mice

All mice were handled in accordance with the guidelines of the Institutional Animal Care and Use Committee of UCSF. Minimal sample sizes were chosen on the basis of standards commonly used in the field and previous experience with similar experiments. All animals of the same genotype and sex were randomly selected for breeding and/or experimentation in this study. Wild-type C57/B6 mice were purchased from Taconic Biosciences and bred in the laboratory. *Igf1*^*f*/*f*^ mice (strain number 012663) and *Cx3cr1*^*CreERt*/+^ mice (strain number 020940) were purchased from The Jackson Laboratory. For timed pregnancy, males and females were paired, and females were observed daily for the presence of a copulation plug. The noon of the day when a plug was observed was noted as embryonic day 0.5. For *Igf1* cKO experiments, 100 mg kg^−1^ of tamoxifen in corn oil was injected intraperitoneally into pregnant dams on embryonic days 11.5 and 12.5. *Igf1*^*f*/*f*^; *Cx3cr1*^*CreERt*/+^ fetuses were used as *Igf1* cKO mice, and their littermates *Igf1*^+/+^; *Cx3cr1*^*CreERt*/+^ and *Igf1*^*f*/*f*^; *Cx3cr1*^+/+^ fetuses were used as controls. During all subsequent experimental procedures, including sample collection, processing, imaging and quantification, the experimenter was blinded to the genotype, sex and age of the mice. Both males and females were included in the mouse experiments. In the EdU labelling experiment, a single dose of EdU (10 mg kg^−1^; provided in the Click-iT EdU Alexa Fluor 647 Imaging Kit from Invitrogen; C10340) was injected intraperitoneally into pregnant mice at embryonic day 14.5. At embryonic days 14.5 and 16.5, the pregnant dams were killed, and the fetal brains were collected, fixed in 4% PFA at 4 °C overnight, cryopreserved in 30% sucrose at 4 °C overnight, embedded in OCT and cryosectioned at 20 μm (EdU labelling and IGF1 staining experiments) or 40 μm (microglia staining experiments) using a Leica cryostat. Additionally, wild-type P5 pups were transcardially perfused with 4% PFA, and their brains were extracted and post-fixed in 4% PFA overnight, cryoprotected in 30% sucrose overnight, embedded in OCT and cryosectioned at 20 μm.

### Human pluripotent stem-cell-derived organoids

The hPSC-derived organoids were generated largely following a previously established protocol^37,38^. In brief, 1323-4 hiPSCs or WA01/H1 and WA09/H9 hESCs were expanded in StemFlex Basal Medium (Gibco; A3349401). After reaching 80% coverage, hPSCs cultured on Matrigel were dissociated into clumps using ReLeSR (STEMCELL Technologies; 100-0483) and equally distributed into a V-bottom 96-well ultra-low-attachment PrimeSurface plate (S-BIO; MS-9096VZ). The rho kinase inhibitor Y-27632 (10 μM) was added during the first 24 h of neural induction to promote survival. Between days 0 and 5, organoids were cultured in neural induction medium (Dulbecco’s modified Eagle medium/F-12, 20% knockout serum, 1% non-essential amino acids, 0.5% GlutaMAX, 0.1 mM β-mercaptoethanol and 1% penicillin–streptomycin) supplemented with the SMAD inhibitors SB431542 (10 μM) and dorsomorphin (5 μM). Between days 6 and 24, organoids were cultured in neural differentiation medium (Neurobasal-A medium, 2% B27 supplement, 1% GlutaMAX and 1% penicillin–streptomycin) supplemented with human recombinant EGF (20 ng ml^−1^) and human recombinant FGF2 (20 ng ml^−1^). Between days 25 and 43, organoids were maintained in neural differentiation medium supplemented with human recombinant brain-derived neurotrophic factor (20 ng ml^−1^) and human recombinant neurotrophin 3 (20 ng ml^−1^). For MGEOs, the media were also supplemented with 5 μM wnt inhibitor IWP-2 on days 4–23, 100 nM smoothened agonist on days 12–23, 100 nM retinoic acid on days 12–15 and 100 nM allopregnanolone on days 16–23 for ventral forebrain patterning. Cortical organoids were not supplemented with IWP-2, smoothened agonist, retinoic acid and allopregnanolone. Each organoid was then moved to six-well plates for long-term culture after week 5. All media and supplements used for organoid cultures were the same as those in a previously published protocol^37,38^.

### Induced microglia

Induced microglial cells were generated from WA01/H1 or WA09/H9 hESC cells using STEMdiff kits, according to the manufacturer’s protocols. In brief, hESCs were differentiated into CD43-expressing haematopoietic progenitor cells for 12 days using a STEMdiff Hematopoietic Kit (STEMCELL; 05310). Haematopoietic progenitor cells were differentiated for 24 days using the STEMdiff Microglia Differentiation Kit (STEMCELL; 100-0019) and matured for an extra 4 days using the STEMdiff Microglia Maturation Kit (STEMCELL; 100-0020) before being added to the organoid cultures for co-culture.

### iMG–organoid engraftment and co-culture

Mature iMG were immediately added to 4-week-old MGE organoids in 96-well ultra-low attachment PrimeSurface plates at 80–100 × 10^3^ microglia per organoid. Trophic factors (100 ng ml^−1^ of IL-34 (PeproTech; 200-34), 25 ng ml^−1^ of CSF1 (PeproTech; 300-25) and 50 ng ml^−1^ TGFβ1 (PeproTech; 100-21)) were added to the culture medium to support microglial survival. One wpt, co-cultured organoid–microglia (neuroimmune organoids) were transferred to a six-well plate and placed on an orbital shaker. The co-cultures were then maintained following the usual organoid maintenance protocol, with the addition of trophic factors.

### Pharmacological manipulation of organoids

Six-week-old organoids were treated with PBS, 100 ng ml^−1^ of recombinant human IGF1 (Abcam; ab269169), 1 μg ml^−1^ of IGF1-neutralizing antibodies (Abcam; ab9572), 1 μM GSK4529 (GSK1904529A; Selleckchem; S1093) or 1 μΜ picropodophyllin (Selleckchem; S7668) for 48 h. Then 10 μΜ BrdU (Abcam; ab142567) was added during the last 4 h to label proliferating cells. Organoids were collected immediately after the treatment for IHC analysis. For the DAPT treatment experiment, PBS or 10 μM DAPT (Abcam; ab120633) was applied to MGEOs transplanted with iMG from 10 to 14 dpt. Organoids were then collected at 14 dpt for IHC analysis.

### Immunohistochemistry

We followed the IHC protocol, as previously reported^14,32^. Human tissue samples were fixed and cryosectioned, as described above. Mouse samples were prepared, as described above in ‘Mice’. Organoids were fixed in 4% PFA for 30–45 min at room temperature and cryopreserved in 30% sucrose in PBS overnight. The organoids were then embedded in OCT and cryosectioned at 14 μm using a Leica cryostat.

The mounted human slides were defrosted overnight at 4 °C and then dried at 37 °C for 3 h. The mounted organoids and mouse slides were dried directly at 37 °C for 30 min. Antigen retrieval was performed for 5–12 min at 95–99 °C using antigen retrieval buffer (BD Pharmingen; 550524). After antigen retrieval, tissue slices were washed with 1× PBS plus 0.1% or 0.3% Triton X-100 and then blocked in blocking buffer (5–10% serum, 1% bovine serum albumin (BSA) and 0.1% Triton X-100 in PBS, or 1% BSA in 0.3% Triton X-100 in PBS) for 1–1.5 h at room temperature before proceeding to incubation with primary antibodies (Supplementary Table 3) overnight at 4 °C. After washing, sections were incubated with species-specific secondary antibodies conjugated to Alexa Fluor dyes (1:500; Invitrogen) for 1.5–2 h at room temperature. For human and embryonic mouse slides, TrueBlack Lipofuscin Autofluorescence Quencher (1:20 in 70% alcohol; Biotium; 23007) was applied for 3–5 min to block autofluorescence. For EdU staining, the EdU working solution was applied to embryonic mouse brain slices after secondary antibody application following the manufacturer’s instructions. Nuclei were counterstained with DAPI (1:1,000 from 1 mg ml^−1^ of stock; Invitrogen; 2031179) for 5 min. Images were captured using a Leica STELLARIS 8 confocal microscope. For organoid experiments, three slices of each organoid were imaged, quantified using ImageJ (1.54) and averaged for the final statistical analysis.

### Three-dimensional reconstruction and image analysis

Three-dimensional reconstructions were generated using the Imaris software (Oxford Instruments). For distance analysis, microglia were reconstructed using surface modules, whereas Ki-67^+^ or DAPI^+^ cells were reconstructed with spot modules. The distance from the centre of each cell (spot) to the nearest microglia (surface) was determined using Imaris. The distance distributions of the Ki-67^+^ and Ki-67^−^ cells to the nearest microglia were calculated accordingly.

### Single-nucleus preparation

Single-nucleus suspensions were prepared from postmortem human samples. About 50 mg of sectioned freshly frozen human brain tissue was homogenized in lysis buffer (0.32 M sucrose, 5 mM CaCl_2_, 3 mM MgAc_2_, 0.1 mM EDTA, 10 mM Tris-HCl, 1 mM dithiothreitol and 0.1% Triton X-100 in diethyl pyrocarbonate-treated water) plus 0.4 U μl^−1^ of RNase inhibitor (Takara; catalogue no. 2313A) on ice. Then, the homogenate was loaded into a 30-ml-thick polycarbonate ultracentrifuge tube (Beckman Coulter; catalogue number 355631), and 9 ml of sucrose cushion solution (1.8 M sucrose, 3 mM MgAc_2_, 1 mM dithiothreitol and 10 mM Tris-HCl in diethyl pyrocarbonate-treated water) was added to the bottom of the tube. The tubes with tissue homogenate and sucrose cushions were then ultracentrifuged at 107,000*g* for 2.5 h at 4 °C. The pellet was recovered in 250-μl ice-cold PBS for 20 min, resuspended in nuclei sorting buffer (PBS, 1% BSA, 0.5 mM EDTA and 0.1 U μl^−1^ of RNase inhibitor) and filtered through a 40-μm cell strainer to obtain single-nucleus suspensions for FACS/fluorescence-activated nucleus sorting.

### Single-cell preparation

Single-cell suspensions of 1323-4 hiPSC-derived organoids were prepared using neural tissue dissociation kits (P) (Miltenyi Biotec; 130-092-628) following the manufacturer’s instructions. In brief, 12–16 organoids per experimental condition were processed through a gentle two-step enzymatic dissociation procedure, as instructed. Five mg ml^−1^ of Actinomycin D (Sigma-Aldrich; A1410), 10 mg ml^−1^ of anisomycin (Sigma-Aldrich; A9789) and 10 mM triptolide (Sigma-Aldrich; T3652) were added before tissue digestion to inhibit the cellular transcriptome. Following digestion, organoids were mechanically triturated using fire-polished glass pipettes, filtered through a 40-μm cell strainer test tube (Corning; 352235), pelleted at 300*g* for 5 min and washed twice with Dulbecco’s phosphate-buffered saline (DPBS) before proceeding to 10× genomics scRNA library preparation. For samples that needed FACS, the single-cell pellet was resuspended in cell sorting buffer (DPBS, 1% BSA and 0.1 U μl^−1^ of RNase inhibitor).

### FACS and fluorescence-activated nucleus sorting

Single-nucleus suspensions from fresh-frozen human samples were stained with antibodies of PU.1 (Cell Signaling Technology; 81886S; 1:100) and OLIG2 (Abcam; ab225100; 1:2,500) overnight at 4 °C. PU.1 and OLIG2 antibodies were conjugated with fluorescence upon purchase. DAPI (1:1,000) was added for 5 min on the second day. Single-cell suspensions from organoids were stained with DAPI (1:1,000) for 5 min in cell sorting buffer (DPBS; 1% BSA and 0.1 U μl^−1^ of RNase inhibitor). The single-nucleus/cell suspension was then centrifuged at 300*g* for 5 min, resuspended in nucleus/cell sorting buffer and filtered through a 40-μm cell strainer for final analysis and sorting using a FACSAria II Cell Sorter (BD Biosciences). Target cells were collected in nucleus/cell sorting buffer for future sequencing library preparation.

### Single-cell and single-nucleus RNA library preparation

Nuclei and cells were counted using a haemocytometer and resuspended to a final concentration of 300–1,000 cells/nuclei per microlitre in PBS. Single-nucleus/cell RNA-seq libraries were prepared using 10× Genomics Chromium Next GEM Single Cell v.3.1 kit according to the manufacturer’s instructions, targeting for 5,000 nuclei/cells per sample. Single-cell/nucleus libraries were then sequenced on the NovaSeq 6000 machine, with a sequencing depth of 50,000 reads per cell.

### Single-cell and single-nucleus RNA-seq data analysis

Sequencing results were then aligned to the GRCh38 genome (gex-GRCh38-2020-A) using Cell Ranger v.6.1.2 (10× Genomics). Then ‘--include-introns’ was used to include premature messenger RNA in single-nucleus samples. Gene counts then underwent a doublet removal step using DoubletFinder v.2.0.3 (https://www.cell.com/cell-systems/fulltext/S2405-4712(19)30073-0).

The output (count matrix) was used as the main input file for all downstream analyses using Seurat v.5.1.0. For human snRNA-seq, nuclei with UMIs of less than 1,000, gene features of less than 1,000 or more than 100,000 or percentage of mitochondrial genes of more than 3% were filtered out. For organoid scRNA-seq, cells with UMIs of less than 800 or more than 50,000, gene features of less than 500 or more than 10,000 or percentage of mitochondrial genes less than 2% or more than 25% were filtered out. For FACS-isolated iMG scRNA-seq, cells with UMIs of less than 1,000 or more than 80,000, gene features of less than 1,000 or more than 20,000 or mitochondrial genes less than 20% were filtered out. MALAT1, mitochondrial genes (MT-), ribosomal protein-encoding genes (RPS- and RPL-) and haemoglobin genes (HB-) were excluded from further analysis. Standard data normalization, variable feature identification, linear transformations, dimensional reduction, UMAP embedding and unsupervised clustering were conducted using the standard Seurat pipeline^35^. Cell-type cluster identification was performed on the basis of the expression of known marker genes, as shown in Extended Data Figs. 2, 7 and 10. For scRNA-seq of GFP-labelled iMG, iMG were purified in silico using canonical microglia/macrophage markers, including *AIF1*, *CX3CR1*, *C3*, *PTPRC*, *ITGAM* and *CD68*.

We analysed cell–cell interaction using CellChat v.2 (ref. ^36^). For development-based analysis, independent CellChat files were generated from ‘embryonic’ and ‘perinatal’ Seurat objects, and a comparison analysis was conducted between them. A heat map was created using GraphPad Prism 9 according to the interacting probability of significant ligand–receptor interactions involved in microglial regulation of interneurons (CIN).

DEG analysis was conducted on the basis of the Seurat-default non-parametric Wilcoxon rank-sum test. Pathways with enriched DEGs were generated using Enrichr (https://maayanlab.cloud/Enrichr/#) on the basis of the Reactome Pathway Database, Kyoto Encyclopedia of Genes and Genomes, GEO and Gene Ontology database. The full names of the pathways shown in Fig. 4 are as follows: IGF1R 46: IGF1R drug inhibition 46 (kinase perturbations from GEO down; GSE14024); IGF1R 52: IGF1R knockdown 52 (kinase perturbations from GEO down; GSE16684); mitotic sister: mitotic sister chromatid segregation (GO:0000070); aerobic electron: aerobic electron transport chain; respiratory: respiratory electron transport, ATP synthesis by chemiosmotic coupling, heat production by uncoupling proteins (R-HSA-163200).

### Principal component analysis

The published sequencing datasets for comparison were collected from eleven previous papers^39,41–50^. The specific papers and corresponding NIH GEO datasets used were as follows: GSE89189 (ref. ^39^); (GSE123021, GSE123022, GSE123024 and GSE123025) (ref. ^41^); GSE121654 (ref. ^42^); GSE141862 (ref. ^43^); (GSE133345 and GSE137010) (ref. ^44^); GSE180945 (ref. ^45^); GSE178317 (ref. ^46^); (GSE139549 and GSE139550) (ref. ^47^); GSE85839 (ref. ^48^); GSE97744 (ref. ^49^); GSE99074 (ref. ^50^). Each dataset was collected, filtered and grouped by appropriate characteristics, including species, real/derived, bulk/single cell, age and protocol details. To facilitate comparison, the groups within each set were pooled into single representations.

Once the data were collected and preprocessed, the pooled samples were processed using Scanpy v.1.10.3 (https://github.com/scverse/scanpy). Specifically, the cells were normalized by total counts over all genes using scanpy.pp.normalize_total. They were then logarithmized using scanpy.pp.log1p. For use in downstream PCA, highly variable genes were calculated using scanpy.pp.highly_variable_genes. The number of top genes was configured to 2,000. In the final steps, PCA was performed using scanpy.tl.pca (default of 50 components), and a scatter plot using the coordinates of PCA 1 and 2 was plotted for each cell representation using scanpy.pl.pca.

### CRISPR–Cas9 gene editing

A WA09/H9 stem cell line with an IGF1 loss-of-function mutation (IGF1 knockout) was generated using CRISPR–Cas9-based non-homology end joining, largely following the protocol of the Alt-R CRISPR–Cas9 System from Integrated DNA Technologies (IDT). The guide RNA (5′TCGTGGATGAGTGCTGCTTC3′) was selected from the predesigned Alt-R CRISPR–Cas9 guide RNA (IDT). Equal amounts of CRISPR RNA and ATTO 550 labelled tracrRNA (IDT; 1075927) were mixed to a final concentration of 100 μΜ, heated to 95 °C for 5 min and then cooled to room temperature for annealing followed by the formation of the ribonucleoprotein complex with Alt-R S.p. HiFi Cas9 Nuclease V3 (IDT; 1081061) at room temperature for 20 min. The ribonucleoprotein complex was delivered to single-stem-cell suspensions using the Neon Electroporation System (1,400 V; 20 ms; one pulse) according to the manufacturer’s instructions. After electroporation, ATTO 550+ cells were selected by FACS after 3 days of culture and sparsely seeded to form a single-cell colony. A loss-of-function mutation cell line was selected by Sanger sequencing with out-of-frame mutations at the target site, followed by exclusion of any mutations at the top 5 potential off-target sites. Further Sanger sequencing confirmation, reverse transcription–quantitative polymerase chain reaction and IHC were performed to confirm IGF1 knockout.

### Time-lapse imaging of microglia–MGE progenitor interactions

To visualize the interactions between engrafted microglia and MGE progenitors in MGEOs, we used MGE organoids generated from NKX2.1-GFP cells and iMG derived from tdTomato-labelled WA09/H9 cells using lentiviral transduction (SignaGen Laboratories; SL100289). Live imaging was performed 2–3 weeks after iMG transplantation. For imaging, engrafted organoids were transferred to a flat glass-bottom six-well plate, with one organoid per well with 500 μl of culture medium. Time-lapse imaging was conducted using a Leica SP8 confocal microscope at 37 °C and 5% CO_2_. *Z*-stacks were captured every 5 min over a 12-h period and processed using maximum intensity projections to visualize dynamic cellular interactions.

### Data analysis, statistics and presentation

For all quantifications, images were acquired and quantified blindly to genotype or treatment. Statistical analyses were performed using GraphPad Prism (v.10.1.0), as shown in each figure legend.

### Reporting summary

Further information on research design is available in the Nature Portfolio Reporting Summary linked to this article.

## Online content

Any methods, additional references, Nature Portfolio reporting summaries, source data, extended data, supplementary information, acknowledgements, peer review information; details of author contributions and competing interests; and statements of data and code availability are available at 10.1038/s41586-025-09362-8.

## Supplementary information

Supplementary Figures Supplementary Figs. 1–4.

Reporting Summary

Supplementary Tables Supplementary Tables 1–3.

Peer Review file

Supplementary Video 1 Time-lapse live recording revealing active interactions between iMG and proliferating progenitors in 6-week-old MGE organoids. iMGs were labelled with tdTomato (red), whereas MGE progenitors and their immediate progeny were visualized using NKX2.1-GFP (green). The recording time is displayed in the top left corner of each image in the format ‘hours:minutes’.

## Source data

Source Data Extended Data Fig. 1

Source Data Extended Data Fig. 15

## Data Availability

All data are available in the main text or Supplementary Information. Raw sequencing datasets and processed Seurat objects were deposited in the Gene Expression Omnibus (accession numbers GSE296073 and GSE274829) and Zenodo (10.5281/zenodo.15299853)^96^, respectively. All the other data are available upon request. Source data are provided with this paper.

## Code availability

The custom code used in the analyses is available at GitHub (https://github.com/DIANKUNYU/R-script-used-for-Yu-2025 and https://github.com/codycollier/mglia-nat25).

## References

[1] Loomba, S. et al. Connectomic comparison of mouse and human cortex. *Science* **377**, eabo0924 (2022). 35737810 10.1126/science.abo0924

[2] Dzaja, D., Hladnik, A., Bicanic, I., Bakovic, M. & Petanjek, Z. Neocortical calretinin neurons in primates: increase in proportion and microcircuitry structure. *Front. Neuroanat.* **8**, 103 (2014). 25309344 10.3389/fnana.2014.00103 PMC4174738

[3] Hornung, J. P. & De Tribolet, N. Distribution of GABA-containing neurons in human frontal cortex: a quantitative immunocytochemical study. *Anat. Embryol.* **189**, 139–145 (1994). 10.1007/BF00185772 8010412

[4] del Rio, M. R. & DeFelipe, J. Colocalization of calbindin D-28k, calretinin, and GABA immunoreactivities in neurons of the human temporal cortex. *J. Comp. Neurol.* **369**, 472–482 (1996). 8743426 10.1002/(SICI)1096-9861(19960603)369:3<472::AID-CNE11>3.0.CO;2-K

[5] Cho, K. K. et al. Gamma rhythms link prefrontal interneuron dysfunction with cognitive inflexibility in *Dlx5/6*^+/−^ mice. *Neuron* **85**, 1332–1343 (2015). 25754826 10.1016/j.neuron.2015.02.019 PMC4503262

[6] Kepecs, A. & Fishell, G. Interneuron cell types are fit to function. *Nature* **505**, 318–326 (2014). 24429630 10.1038/nature12983 PMC4349583

[7] Lauber, E., Filice, F. & Schwaller, B. Parvalbumin neurons as a hub in autism spectrum disorders. *J. Neurosci. Res.* **96**, 360–361 (2018). 29271051 10.1002/jnr.24204

[8] Sohal, V. S. & Rubenstein, J. L. R. Excitation–inhibition balance as a framework for investigating mechanisms in neuropsychiatric disorders. *Mol. Psychiatry* **24**, 1248–1257 (2019). 31089192 10.1038/s41380-019-0426-0 PMC6742424

[9] Chen, S. F. et al. Serum levels of brain-derived neurotrophic factor and insulin-like growth factor 1 are associated with autonomic dysfunction and impaired cerebral autoregulation in patients with epilepsy. *Front. Neurol.* **9**, 969 (2018). 30524358 10.3389/fneur.2018.00969 PMC6256185

[10] Nakazawa, K. et al. GABAergic interneuron origin of schizophrenia pathophysiology. *Neuropharmacology* **62**, 1574–1583 (2012). 21277876 10.1016/j.neuropharm.2011.01.022 PMC3090452

[11] Hansen, D. V. et al. Non-epithelial stem cells and cortical interneuron production in the human ganglionic eminences. *Nat. Neurosci.* **16**, 1576–1587 (2013). 24097039 10.1038/nn.3541 PMC4191718

[12] Ma, T. et al. Subcortical origins of human and monkey neocortical interneurons. *Nat. Neurosci.* **16**, 1588–1597 (2013). 24097041 10.1038/nn.3536

[13] Chen, J., Choi, J. J., Lin, P. Y. & Huang, E. J. Pathogenesis of germinal matrix hemorrhage: insights from single-cell transcriptomics. *Annu. Rev. Pathol.* **20**, 221–243 (2025). 39401848 10.1146/annurev-pathmechdis-111523-023446 PMC11759652

[14] Paredes, M. F. et al. Nests of dividing neuroblasts sustain interneuron production for the developing human brain. *Science* **375**, eabk2346 (2022). 35084970 10.1126/science.abk2346 PMC8887556

[15] Paredes, M. F. et al. Extensive migration of young neurons into the infant human frontal lobe. *Science*10.1126/science.aaf7073 (2016). 10.1126/science.aaf7073 PMC5436574 27846470

[16] Monier, A. et al. Entry and distribution of microglial cells in human embryonic and fetal cerebral cortex. *J. Neuropathol. Exp. Neurol.* **66**, 372–382 (2007). 17483694 10.1097/nen.0b013e3180517b46

[17] Ribeiro Xavier, A. L., Kress, B. T., Goldman, S. A., Lacerda de Menezes, J. R. & Nedergaard, M. A distinct population of microglia supports adult neurogenesis in the subventricular zone. *J. Neurosci.* **35**, 11848–11861 (2015). 26311768 10.1523/JNEUROSCI.1217-15.2015 PMC4549398

[18] Fantin, A. et al. Tissue macrophages act as cellular chaperones for vascular anastomosis downstream of VEGF-mediated endothelial tip cell induction. *Blood* **116**, 829–840 (2010). 20404134 10.1182/blood-2009-12-257832 PMC2938310

[19] Chen, J. et al. Proinflammatory immune cells disrupt angiogenesis and promote germinal matrix hemorrhage in prenatal human brain. *Nat. Neurosci.* **27**, 2115–2129 (2024). 39349662 10.1038/s41593-024-01769-2 PMC11537974

[20] Seo, Y. et al. Excessive microglial activation aggravates olfactory dysfunction by impeding the survival of newborn neurons in the olfactory bulb of Niemann–Pick disease type C1 mice. *Biochim. Biophys. Acta* **1842**, 2193–2203 (2014). 25132229 10.1016/j.bbadis.2014.08.005

[21] Giera, S. et al. Microglial transglutaminase-2 drives myelination and myelin repair via GPR56/ADGRG1 in oligodendrocyte precursor cells. *eLife* **7**, e33385 (2018). 29809138 10.7554/eLife.33385 PMC5980231

[22] McNamara, N. B. et al. Microglia regulate central nervous system myelin growth and integrity. *Nature* **613**, 120–129 (2023). 36517604 10.1038/s41586-022-05534-y PMC9812791

[23] Hagemeyer, N. et al. Microglia contribute to normal myelinogenesis and to oligodendrocyte progenitor maintenance during adulthood. *Acta Neuropathol.* **134**, 441–458 (2017). 28685323 10.1007/s00401-017-1747-1 PMC5951721

[24] Miron, V. E. et al. M2 microglia and macrophages drive oligodendrocyte differentiation during CNS remyelination. *Nat. Neurosci.* **16**, 1211–1218 (2013). 23872599 10.1038/nn.3469 PMC3977045

[25] Parkhurst, C. N. et al. Microglia promote learning-dependent synapse formation through brain-derived neurotrophic factor. *Cell* **155**, 1596–1609 (2013). 24360280 10.1016/j.cell.2013.11.030 PMC4033691

[26] Li, T. et al. A splicing isoform of GPR56 mediates microglial synaptic refinement via phosphatidylserine binding. *EMBO J.* **39**, e104136 (2020). 32452062 10.15252/embj.2019104136 PMC7429740

[27] Schafer, D. P. et al. Microglia sculpt postnatal neural circuits in an activity and complement-dependent manner. *Neuron* **74**, 691–705 (2012). 22632727 10.1016/j.neuron.2012.03.026 PMC3528177

[28] Lehrman, E. K. et al. CD47 protects synapses from excess microglia-mediated pruning during development. *Neuron* **100**, 120–134 (2018). 30308165 10.1016/j.neuron.2018.09.017 PMC6314207

[29] Scott-Hewitt, N. et al. Local externalization of phosphatidylserine mediates developmental synaptic pruning by microglia. *EMBO J.* **39**, e105380 (2020). 32657463 10.15252/embj.2020105380 PMC7429741

[30] Paolicelli, R. C. et al. Synaptic pruning by microglia is necessary for normal brain development. *Science* **333**, 1456–1458 (2011). 21778362 10.1126/science.1202529

[31] Squarzoni, P. et al. Microglia modulate wiring of the embryonic forebrain. *Cell Rep.* **8**, 1271–1279 (2014). 25159150 10.1016/j.celrep.2014.07.042

[32] Yu, D. et al. Microglial GPR56 is the molecular target of maternal immune activation-induced parvalbumin-positive interneuron deficits. *Sci. Adv.* **8**, eabm2545 (2022). 35544642 10.1126/sciadv.abm2545 PMC9075805

[33] Saunders, A. et al. Molecular diversity and specializations among the cells of the adult mouse brain. *Cell* **174**, 1015–1030 (2018). 30096299 10.1016/j.cell.2018.07.028 PMC6447408

[34] Nott, A., Schlachetzki, J. C. M., Fixsen, B. R. & Glass, C. K. Nuclei isolation of multiple brain cell types for omics interrogation. *Nat. Protoc.* **16**, 1629–1646 (2021). 33495627 10.1038/s41596-020-00472-3 PMC7969463

[35] Hao, Y. et al. Dictionary learning for integrative, multimodal and scalable single-cell analysis. *Nat. Biotechnol.* **42**, 293–304 (2024). 37231261 10.1038/s41587-023-01767-y PMC10928517

[36] Jin, S. et al. Inference and analysis of cell-cell communication using CellChat. *Nat. Commun.* **12**, 1088 (2021). 33597522 10.1038/s41467-021-21246-9 PMC7889871

[37] Sloan, S. A., Andersen, J., Pasca, A. M., Birey, F. & Pasca, S. P. Generation and assembly of human brain region-specific three-dimensional cultures. *Nat. Protoc.* **13**, 2062–2085 (2018). 30202107 10.1038/s41596-018-0032-7 PMC6597009

[38] Birey, F. et al. Assembly of functionally integrated human forebrain spheroids. *Nature* **545**, 54–59 (2017). 28445465 10.1038/nature22330 PMC5805137

[39] Abud, E. M. et al. iPSC-derived human microglia-like cells to study neurological diseases. *Neuron* **94**, 278–293 (2017). 28426964 10.1016/j.neuron.2017.03.042 PMC5482419

[40] Schafer, S. T. et al. An in vivo neuroimmune organoid model to study human microglia phenotypes. *Cell* **186**, 2111–2126 (2023). 37172564 10.1016/j.cell.2023.04.022 PMC10284271

[41] Li, Q. et al. Developmental heterogeneity of microglia and brain myeloid cells revealed by deep single-cell RNA sequencing. *Neuron* **101**, 207–223 (2019). 30606613 10.1016/j.neuron.2018.12.006 PMC6336504

[42] Hammond, T. R. et al. Single-cell RNA sequencing of microglia throughout the mouse lifespan and in the injured brain reveals complex cell-state changes. *Immunity* **50**, 253–271 (2019). 30471926 10.1016/j.immuni.2018.11.004 PMC6655561

[43] Kracht, L. et al. Human fetal microglia acquire homeostatic immune-sensing properties early in development. *Science* **369**, 530–537 (2020). 32732419 10.1126/science.aba5906

[44] Bian, Z. et al. Deciphering human macrophage development at single-cell resolution. *Nature* **582**, 571–576 (2020). 32499656 10.1038/s41586-020-2316-7

[45] Popova, G. et al. Human microglia states are conserved across experimental models and regulate neural stem cell responses in chimeric organoids. *Cell Stem Cell* **28**, 2153–2166 (2021). 34536354 10.1016/j.stem.2021.08.015 PMC8642295

[46] Drager, N. M. et al. A CRISPRi/a platform in human iPSC-derived microglia uncovers regulators of disease states. *Nat. Neurosci.* **25**, 1149–1162 (2022). 35953545 10.1038/s41593-022-01131-4 PMC9448678

[47] Guttikonda, S. R. et al. Fully defined human pluripotent stem cell-derived microglia and tri-culture system model C3 production in Alzheimer’s disease. *Nat. Neurosci.* **24**, 343–354 (2021). 33558694 10.1038/s41593-020-00796-z PMC8382543

[48] Muffat, J. et al. Efficient derivation of microglia-like cells from human pluripotent stem cells. *Nat. Med.* **22**, 1358–1367 (2016). 27668937 10.1038/nm.4189 PMC5101156

[49] Douvaras, P. et al. Directed differentiation of human pluripotent stem cells to microglia. *Stem Cell Rep.* **8**, 1516–1524 (2017). 10.1016/j.stemcr.2017.04.023 PMC5470097 28528700

[50] Galatro, T. F. et al. Transcriptomic analysis of purified human cortical microglia reveals age-associated changes. *Nat. Neurosci.* **20**, 1162–1171 (2017). 28671693 10.1038/nn.4597

[51] Bennett, F. C. et al. A combination of ontogeny and CNS environment establishes microglial identity. *Neuron* **98**, 1170–1183 (2018). 29861285 10.1016/j.neuron.2018.05.014 PMC6023731

[52] Park, D. S. et al. iPS-cell-derived microglia promote brain organoid maturation via cholesterol transfer. *Nature* **623**, 397–405 (2023). 37914940 10.1038/s41586-023-06713-1

[53] Menassa, D. A. et al. The spatiotemporal dynamics of microglia across the human lifespan. *Dev. Cell* **57**, 2127–2139 (2022). 35977545 10.1016/j.devcel.2022.07.015 PMC9616795

[54] Ciceri, G. et al. An epigenetic barrier sets the timing of human neuronal maturation. *Nature* **626**, 881–890 (2024). 38297124 10.1038/s41586-023-06984-8 PMC10881400

[55] Sabate-Soler, S. et al. Microglia integration into human midbrain organoids leads to increased neuronal maturation and functionality. *Glia* **70**, 1267–1288 (2022). 35262217 10.1002/glia.24167 PMC9314680

[56] Goulburn, A. L. et al. A targeted NKX2.1 human embryonic stem cell reporter line enables identification of human basal forebrain derivatives. *Stem Cells* **29**, 462–473 (2011). 21425409 10.1002/stem.587

[57] Kang, H. J. et al. BRCA1 negatively regulates IGF-1 expression through an estrogen-responsive element-like site. *Cell Death Dis.* **3**, e336 (2012). 22739988 10.1038/cddis.2012.78 PMC3388245

[58] Li, H. et al. Disrupting AGR2/IGF1 paracrine and reciprocal signaling for pancreatic cancer therapy. *Cell Rep. Med.* **6**, 101927 (2025). 39914384 10.1016/j.xcrm.2024.101927 PMC11866503

[59] Gao, F. et al. Hedgehog-responsive PDGFRa(+) fibroblasts maintain a unique pool of alveolar epithelial progenitor cells during alveologenesis. *Cell Rep.* **39**, 110608 (2022). 35385750 10.1016/j.celrep.2022.110608 PMC9199394

[60] Lawrence, A. R. et al. Microglia maintain structural integrity during fetal brain morphogenesis. *Cell* **187**, 962–980 (2024). 38309258 10.1016/j.cell.2024.01.012 PMC10869139

[61] Geirsdottir, L. et al. Cross-species single-cell analysis reveals divergence of the primate microglia program. *Cell* **179**, 1609–1622 (2019). 31835035 10.1016/j.cell.2019.11.010

[62] Hu, J. S., Vogt, D., Sandberg, M. & Rubenstein, J. L. Cortical interneuron development: a tale of time and space. *Development* **144**, 3867–3878 (2017). 29089360 10.1242/dev.132852 PMC5702067

[63] Mir, S., Cai, W., Carlson, S. W., Saatman, K. E. & Andres, D. A. IGF-1 mediated neurogenesis involves a novel RIT1/Akt/Sox2 cascade. *Sci. Rep.* **7**, 3283 (2017). 28607354 10.1038/s41598-017-03641-9 PMC5468318

[64] Dempsey, R. J., Sailor, K. A., Bowen, K. K., Tureyen, K. & Vemuganti, R. Stroke-induced progenitor cell proliferation in adult spontaneously hypertensive rat brain: effect of exogenous IGF-1 and GDNF. *J. Neurochem.* **87**, 586–597 (2003). 14535942 10.1046/j.1471-4159.2003.02022.x

[65] Arsenijevic, Y., Weiss, S., Schneider, B. & Aebischer, P. Insulin-like growth factor-I is necessary for neural stem cell proliferation and demonstrates distinct actions of epidermal growth factor and fibroblast growth factor-2. *J. Neurosci.* **21**, 7194–7202 (2001). 11549730 10.1523/JNEUROSCI.21-18-07194.2001 PMC6762999

[66] Abuzzahab, M. J. et al. IGF-I receptor mutations resulting in intrauterine and postnatal growth retardation. *N. Engl. J. Med.* **349**, 2211–2222 (2003). 14657428 10.1056/NEJMoa010107

[67] Juanes, M. et al. Three novel IGF1R mutations in microcephalic patients with prenatal and postnatal growth impairment. *Clin. Endocrinol. (Oxf.)* **82**, 704–711 (2015). 25040157 10.1111/cen.12555

[68] Hodge, R. D., D’Ercole, A. J. & O’Kusky, J. R. Insulin-like growth factor-I (IGF-I) inhibits neuronal apoptosis in the developing cerebral cortex in vivo. *Int. J. Dev. Neurosci.* **25**, 233–241 (2007). 17459648 10.1016/j.ijdevneu.2007.03.004 PMC2255566

[69] Beck, K. D., Powell-Braxton, L., Widmer, H. R., Valverde, J. & Hefti, F. *Igf1* gene disruption results in reduced brain size, CNS hypomyelination, and loss of hippocampal granule and striatal parvalbumin-containing neurons. *Neuron* **14**, 717–730 (1995). 7718235 10.1016/0896-6273(95)90216-3

[70] Carson, M. J., Behringer, R. R., Brinster, R. L. & McMorris, F. A. Insulin-like growth factor I increases brain growth and central nervous system myelination in transgenic mice. *Neuron* **10**, 729–740 (1993). 8386530 10.1016/0896-6273(93)90173-o

[71] Supeno, N. E. et al. IGF-1 acts as controlling switch for long-term proliferation and maintenance of EGF/FGF-responsive striatal neural stem cells. *Int. J. Med. Sci.* **10**, 522–531 (2013). 23532711 10.7150/ijms.5325 PMC3607237

[72] Wamaitha, S. E. et al. IGF1-mediated human embryonic stem cell self-renewal recapitulates the embryonic niche. *Nat. Commun.* **11**, 764 (2020). 32034154 10.1038/s41467-020-14629-x PMC7005693

[73] Han, C. Z. et al. Human microglia maturation is underpinned by specific gene regulatory networks. *Immunity* **56**, 2152–2171 (2023). 37582369 10.1016/j.immuni.2023.07.016 PMC10529991

[74] Arno, B. et al. Neural progenitor cells orchestrate microglia migration and positioning into the developing cortex. *Nat. Commun.* **5**, 5611 (2014). 25425146 10.1038/ncomms6611

[75] Antony, J. M., Paquin, A., Nutt, S. L., Kaplan, D. R. & Miller, F. D. Endogenous microglia regulate development of embryonic cortical precursor cells. *J. Neurosci. Res.* **89**, 286–298 (2011). 21259316 10.1002/jnr.22533

[76] Yamamiya, M., Tanabe, S. & Muramatsu, R. Microglia promote the proliferation of neural precursor cells by secreting osteopontin. *Biochem. Biophys. Res. Commun.* **513**, 841–845 (2019). 31003770 10.1016/j.bbrc.2019.04.076

[77] Cunningham, C. L., Martinez-Cerdeno, V. & Noctor, S. C. Microglia regulate the number of neural precursor cells in the developing cerebral cortex. *J. Neurosci.* **33**, 4216–4233 (2013). 23467340 10.1523/JNEUROSCI.3441-12.2013 PMC3711552

[78] Ferron, S. R. et al. Differential genomic imprinting regulates paracrine and autocrine roles of IGF2 in mouse adult neurogenesis. *Nat. Commun.* **6**, 8265 (2015). 26369386 10.1038/ncomms9265 PMC4579569

[79] Lehtinen, M. K. et al. The cerebrospinal fluid provides a proliferative niche for neural progenitor cells. *Neuron* **69**, 893–905 (2011). 21382550 10.1016/j.neuron.2011.01.023 PMC3085909

[80] Rosin, J. M. et al. Embryonic microglia interact with hypothalamic radial glia during development and upregulate the TAM receptors MERTK and AXL following an insult. *Cell Rep.* **34**, 108587 (2021). 33406432 10.1016/j.celrep.2020.108587

[81] McKinsey, G. L. et al. Radial glia integrin avb8 regulates cell autonomous microglial TGFβ1 signaling that is necessary for microglial identity. *Nat. Commun.* **16**, 2840 (2025). 40121230 10.1038/s41467-025-57684-y PMC11929771

[82] Lancaster, M. A. et al. Cerebral organoids model human brain development and microcephaly. *Nature* **501**, 373–379 (2013). 23995685 10.1038/nature12517 PMC3817409

[83] Mariani, J. et al. Modeling human cortical development in vitro using induced pluripotent stem cells. *Proc. Natl Acad. Sci. USA* **109**, 12770–12775 (2012). 22761314 10.1073/pnas.1202944109 PMC3411972

[84] Pollen, A. A. et al. Establishing cerebral organoids as models of human-specific brain evolution. *Cell* **176**, 743–756 (2019). 30735633 10.1016/j.cell.2019.01.017 PMC6544371

[85] Velasco, S. et al. Individual brain organoids reproducibly form cell diversity of the human cerebral cortex. *Nature* **570**, 523–527 (2019). 31168097 10.1038/s41586-019-1289-x PMC6906116

[86] Qian, X. et al. Brain-region-specific organoids using mini-bioreactors for modeling ZIKV exposure. *Cell* **165**, 1238–1254 (2016). 27118425 10.1016/j.cell.2016.04.032 PMC4900885

[87] Xiang, Y. et al. Fusion of regionally specified hPSC-derived organoids models human brain development and interneuron migration. *Cell Stem Cell* **21**, 383–398 (2017). 28757360 10.1016/j.stem.2017.07.007 PMC5720381

[88] Chen, H. I., Song, H. & Ming, G. L. Applications of human brain oganoids to clinical problems. *Dev. Dyn.* **248**, 53–64 (2019). 30091290 10.1002/dvdy.24662 PMC6312736

[89] Riikonen, R. et al. Cerebrospinal fluid insulin-like growth factors IGF-1 and IGF-2 in infantile autism. *Dev. Med. Child Neurol*. **48**, 751–755 (2006). 16904022 10.1017/S0012162206001605

[90] Vanhala, R., Turpeinen, U. & Riikonen, R. Low levels of insulin-like growth factor-I in cerebrospinal fluid in children with autism. *Dev. Med. Child Neurol.* **43**, 614–616 (2001). 11570630 10.1017/s0012162201001116

[91] Linker, S. B., Mendes, A. P. D. & Marchetto, M. C. IGF-1 treatment causes unique transcriptional response in neurons from individuals with idiopathic autism. *Mol. Autism* **11**, 55 (2020). 32591005 10.1186/s13229-020-00359-w PMC7320548

[92] Tropea, D. et al. Partial reversal of Rett syndrome-like symptoms in MeCP2 mutant mice. *Proc. Natl Acad. Sci. USA* **106**, 2029–2034 (2009). 19208815 10.1073/pnas.0812394106 PMC2644158

[93] Shcheglovitov, A. et al. SHANK3 and IGF1 restore synaptic deficits in neurons from 22q13 deletion syndrome patients. *Nature* **503**, 267–271 (2013). 24132240 10.1038/nature12618 PMC5559273

[94] Yuan, Z. F. et al. Insulin-like growth factor-1 down-regulates the phosphorylation of FXYD1 and rescues behavioral deficits in a mouse model of Rett syndrome. *Front. Neurosci.* **14**, 20 (2020). 32063830 10.3389/fnins.2020.00020 PMC7000522

[95] Castro, J. et al. Functional recovery with recombinant human IGF1 treatment in a mouse model of Rett syndrome. *Proc. Natl Acad. Sci. USA* **111**, 9941–9946 (2014). 24958891 10.1073/pnas.1311685111 PMC4103342

[96] Yu, D., Piao, X. & Wangzhou, A. Microglia regulate GABAergic neurogenesis in prenatal human brain through IGF1. *Zenodo*10.5281/zenodo.15299853 (2025). 10.1038/s41586-025-09362-8 PMC12527950 40770097

