# Supplementary material for: Microglia regulate GABAergic neurogenesis in prenatal human brain through IGF1
Source: Nature. 2025 Aug 6;646(8085):676–86. doi: 10.1038/s41586-025-09362-8 (PMC12527950; doi:10.1038/s41586-025-09362-8)
Supplement: Supplementary file 1 — Supplementary Figs. 1–4. [file 41586_2025_9362_MOESM1_ESM.zip › 2024-08-17269B-s1/SupplementaryFigureLegends.docx]

**Supplementary information**

**Microglia regulate GABAergic neurogenesis in prenatal human brain via IGF1**

Authors: Diankun Yu^1, 11,^ *, Samhita Jain^1, 2, 11^, Andi Wangzhou^1^, Beika Zhu^1^, Wenyuan Shao^1^, Elena J Coley-O'Rourke^1^, Stacy De Florencio^1^, Jae Yeon Kim^1,3,4^, Jennifer Ja-Yoon Choi^5^, Mercedes F Paredes^1,3,4^, Tomasz J Nowakowski^1,3,6,7,8^, Eric J Huang^1,3,5,9, †^, Xianhua Piao^1,2,3,10,^*

**Table of contents:**

Supplementary Fig. 1 | UMAP plots showing cell clusters composition for different enrichment strategies from each sample. a, UMAP plots with cell types of the clusters annotated. b, UMAP plots of each sample according to enrichment strategies.

**Supplementary Fig. 2 | Cell-cell communication pathways revealed by CellChat analysis. a**, Circle plots showing the number of interactions and interaction weights/strength among different types of cells. Arrowed lines depict the direction of signaling, originating from source cell types and targeting recipient cell types. Line thickness corresponds to the number or weights/strength of interactions. **b**, Heatmaps showing the significant signaling pathways in terms of outgoing, incoming, and overall signaling. The color indicates the relative strength of signaling across cell types. The top bar plot displays the total signaling strength for each cell type, while the right bar plot shows the total signaling strength of each signaling pathway across all cell types.

**Supplementary Fig. 3 | Differential cell-cell communication pathways at embryonic and perinatal stages. a**, Circle plots and **b**, heatmaps showing the number and weights/strength of differential interactions between embryonic and perinatal stages. Red color represents increased signaling number/strength in the perinatal stage in comparison to the embryonic stage. **c**-**e**, Heatmaps showing signaling pathways in each cell type at embryonic and perinatal stages in terms of incoming (c), outgoing (d), and overall signaling (e). The listed pathways are signaling pathways that display significantly differential signaling patterns between the embryonic and perinatal stages.

**Supplementary Fig. 4 | The promotion effects of iMG on MGE progenitor proliferation are conserved in iMG and MGEO derived from different hPSC.** The density of NKX2.1^+^Ki-67^+^ proliferating MGE progenitors are significantly increased in (**a**) MGEOs derived from 1323-4 hiPSC transplanted with iMG induced from H1 hESC, (**b**) MGEOs derived from H1 hESC transplanted with iMG induced from H9 hESC, and (**c**) MGEOs derived from H9 hESC transplanted with iMG induced from H9 hESC in addition to MGEOs derived from 1323-4 hiPSC MGEO transplanted with iMG induced from H9 hESC (Fig. 4g, h). N=6,8 in (a); N=5,3 in (b), N=3,4 in (c); upaired two-tailed t test; data were shown as means ± SEM.
