# Supplementary figures and images for: Microglia regulate GABAergic neurogenesis in prenatal human brain through IGF1
Source: Nature. 2025 Aug 6;646(8085):676–86. doi: 10.1038/s41586-025-09362-8 (PMC12527950; doi:10.1038/s41586-025-09362-8)

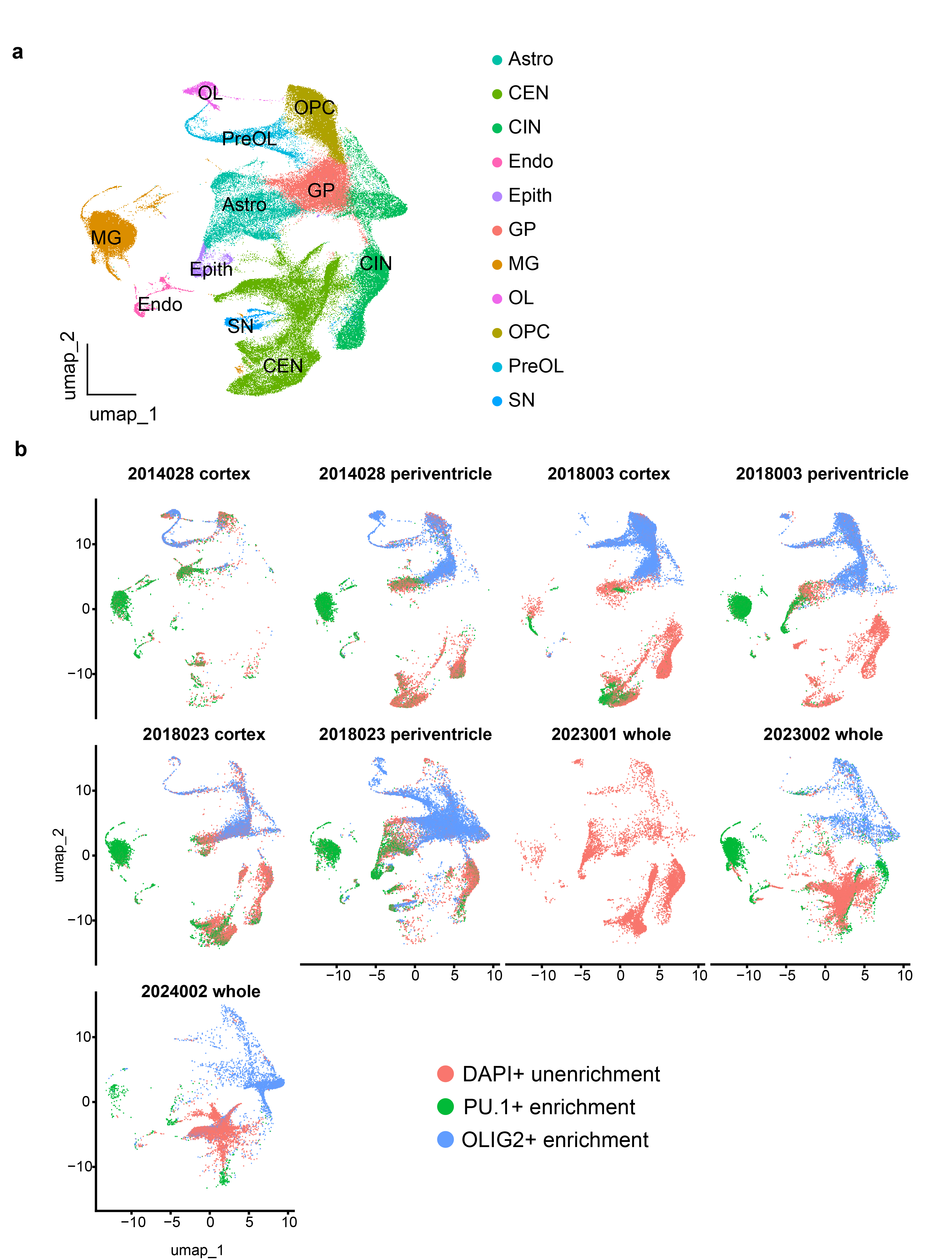

Supplement: Supplementary file 1 — Supplementary Figs. 1–4. [file 41586_2025_9362_MOESM1_ESM.zip › 2024-08-17269B-s1/2024-08-17269B-s1/Supplementary_Fig._1.jpg]

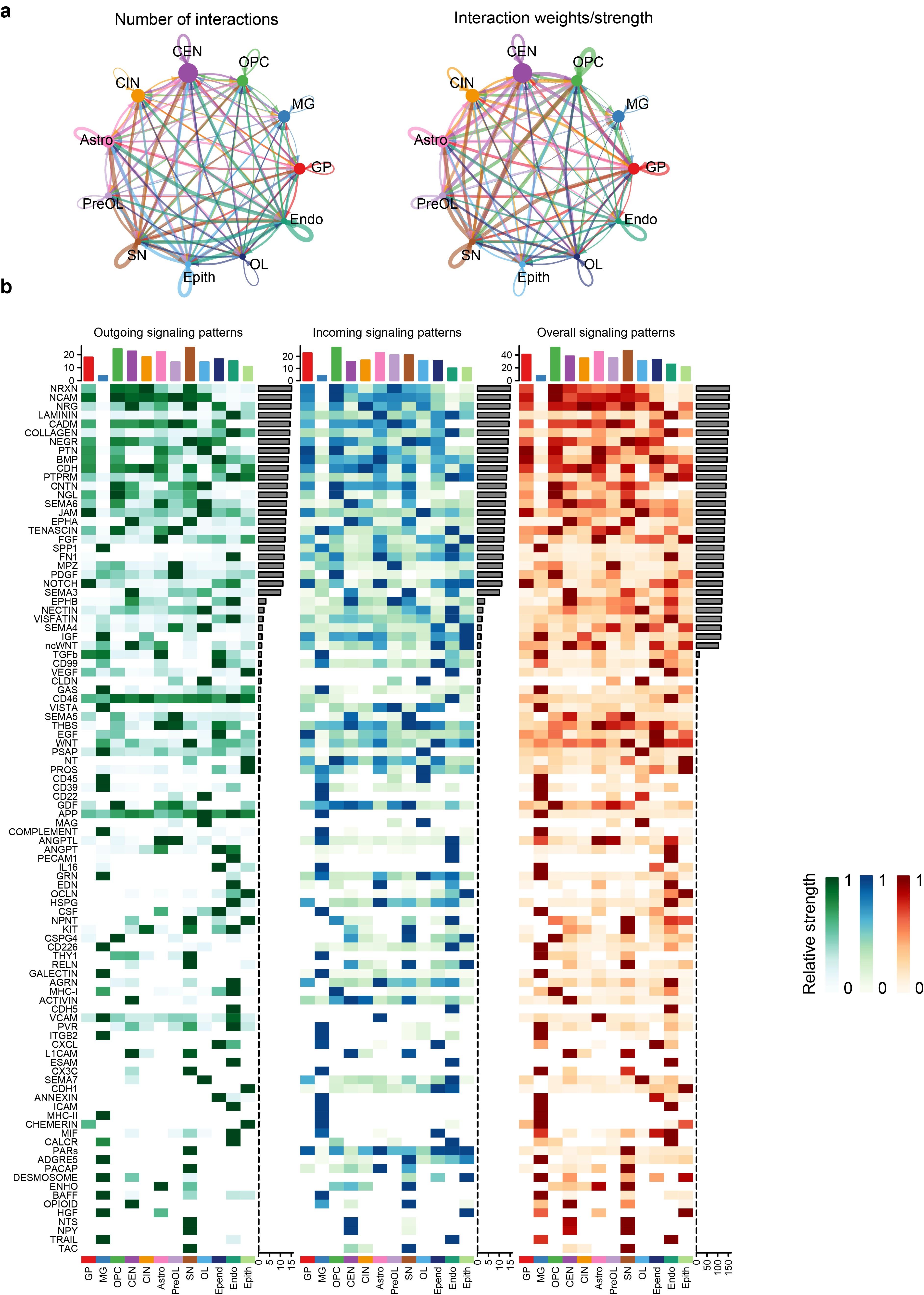

Supplement: Supplementary file 1 — Supplementary Figs. 1–4. [file 41586_2025_9362_MOESM1_ESM.zip › 2024-08-17269B-s1/2024-08-17269B-s1/Supplementary_Fig._2.jpg]

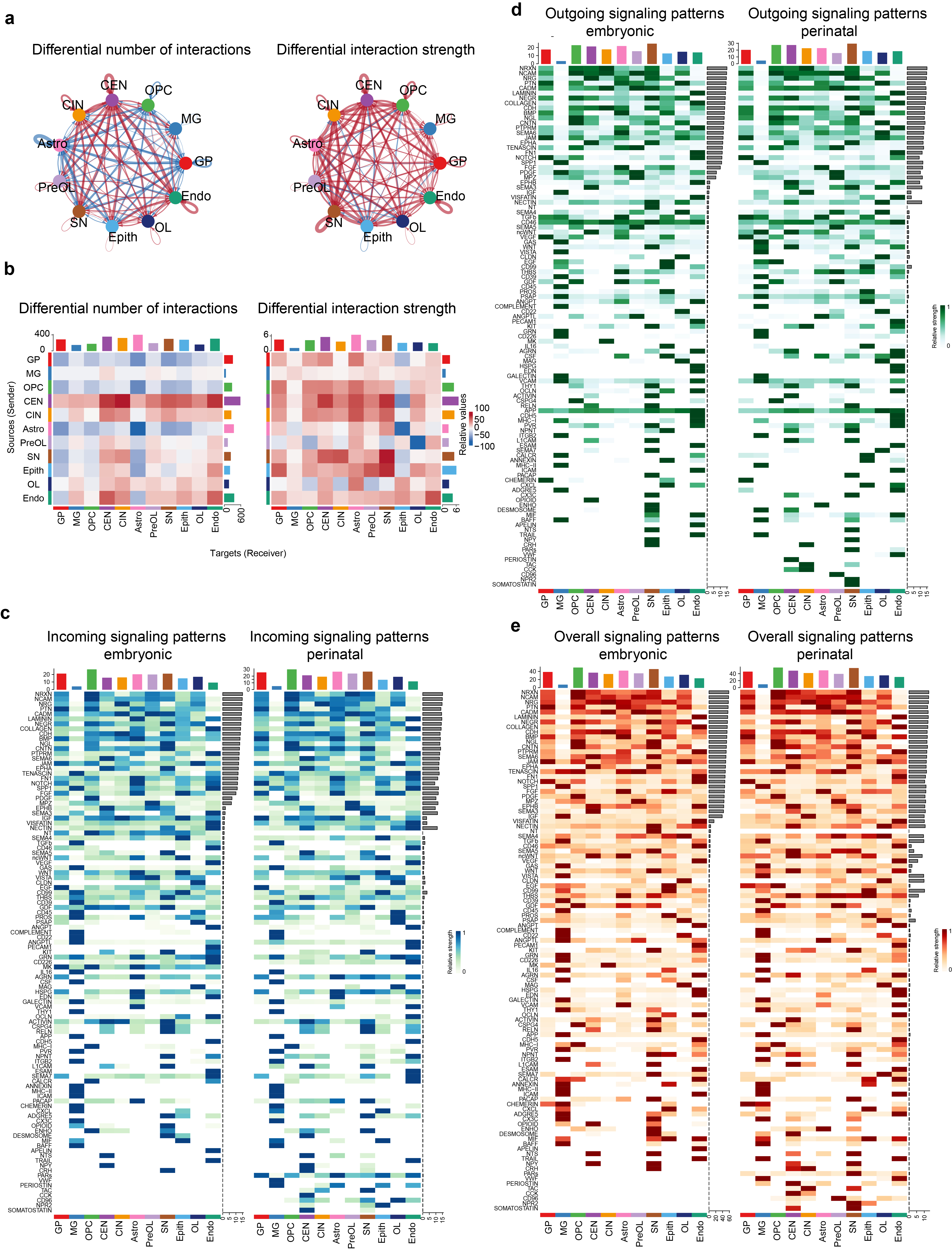

Supplement: Supplementary file 1 — Supplementary Figs. 1–4. [file 41586_2025_9362_MOESM1_ESM.zip › 2024-08-17269B-s1/2024-08-17269B-s1/Supplementary_Fig._3.jpg]

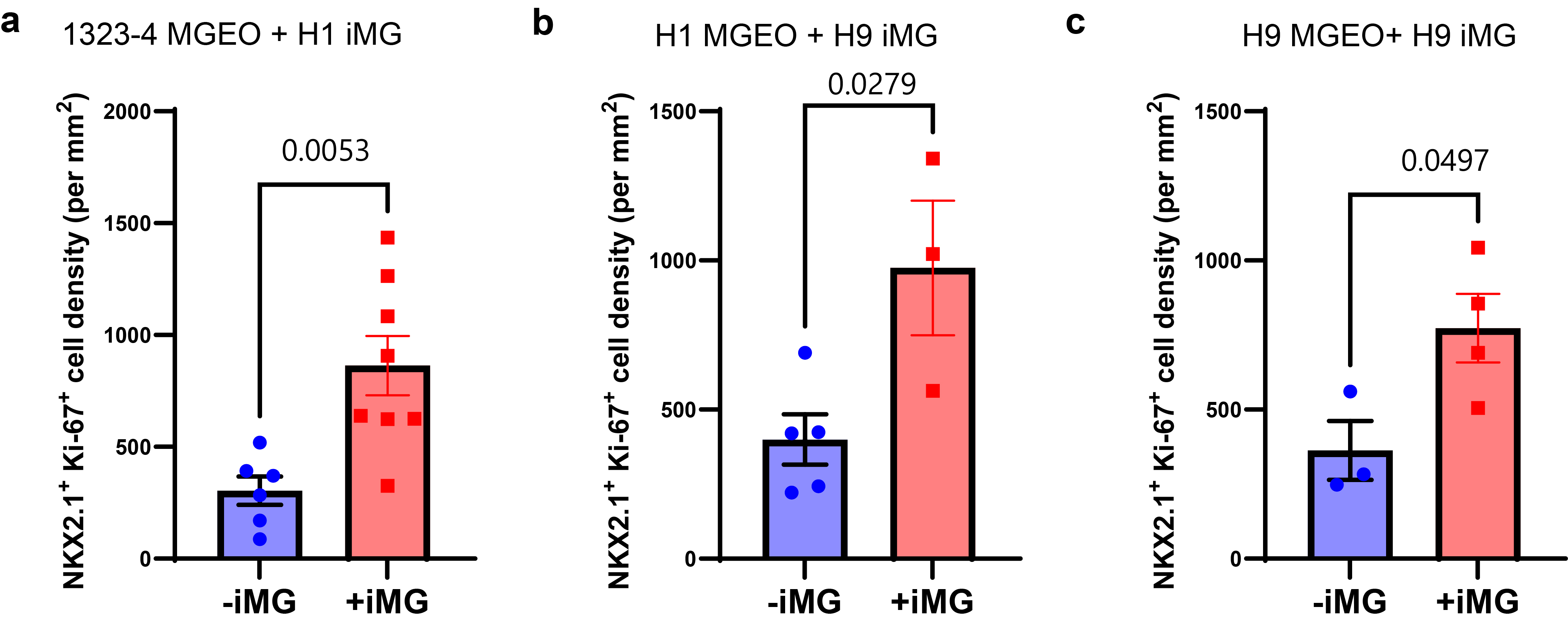

Supplement: Supplementary file 1 — Supplementary Figs. 1–4. [file 41586_2025_9362_MOESM1_ESM.zip › 2024-08-17269B-s1/2024-08-17269B-s1/Supplementary_Fig._4.jpg]
