# Supplementary material for: Microglia regulate GABAergic neurogenesis in prenatal human brain through IGF1
Source: Nature. 2025 Aug 6;646(8085):676–86. doi: 10.1038/s41586-025-09362-8 (PMC12527950; doi:10.1038/s41586-025-09362-8)
Supplement: Supplementary file 2 — Reporting Summary [file 41586_2025_9362_MOESM2_ESM.pdf]

nature portfolio

Corresponding author(s): Diankun Yu, Xianhua Piao

Last updated by author(s): Jun 18, 2025

Reporting Summary

Nature Portfolio wishes to improve the reproducibility of the work that we publish. This form provides structure for consistency and transparency in reporting. For further information on Nature Portfolio policies, see our Editorial Policies and the Editorial Policy Checklist.

Statistics

For all statistical analyses, confirm that the following items are present in the figure legend, table legend, main text, or Methods section.

n/a	Confirmed	
☐	☒	The exact sample size (*n*) for each experimental group/condition, given as a discrete number and unit of measurement
☐	☒	A statement on whether measurements were taken from distinct samples or whether the same sample was measured repeatedly
☐	☒	The statistical test(s) used AND whether they are one- or two-sided<br>*Only common tests should be described solely by name; describe more complex techniques in the Methods section.*
☐	☒	A description of all covariates tested
☐	☒	A description of any assumptions or corrections, such as tests of normality and adjustment for multiple comparisons
☐	☒	A full description of the statistical parameters including central tendency (e.g. means) or other basic estimates (e.g. regression coefficient) AND variation (e.g. standard deviation) or associated estimates of uncertainty (e.g. confidence intervals)
☐	☒	For null hypothesis testing, the test statistic (e.g. *F*, *t*, *r*) with confidence intervals, effect sizes, degrees of freedom and *P* value noted<br>*Give P values as exact values whenever suitable.*
☒	☐	For Bayesian analysis, information on the choice of priors and Markov chain Monte Carlo settings
☒	☐	For hierarchical and complex designs, identification of the appropriate level for tests and full reporting of outcomes
☒	☐	Estimates of effect sizes (e.g. Cohen's *d*, Pearson's *r*), indicating how they were calculated

Our web collection on statistics for biologists contains articles on many of the points above.

Software and code

Policy information about availability of computer code

Data collection	Leica Application Suite X (4.7.0.28176) was used for microscopic images collection; BD FACSDiva (9.0.1) was used for FACS data collection;
Data analysis	Leica LAX (3.7.25997.6), Image J (1.54), and Imaris (9.8.2) used for IHC images analysis and presentation. CellRanger (v6.1.2), Rstudio (2024.09.00) and Scanpy (1.10.3)were used for seq data analysis. Graphpad v10 was used for statistical analysis and data presentation. FACS data were analyzed by Floreada.io (https://floreada.io/analysis)<br>Code availability: Zenodo (DOI: 10.5281/zenodo.15299853, https://zenodo.org/records/15299853);<br>Github (https://github.com/DIANKUNYU/R-script-used-for-Yu-2025 and https://github.com/codycollier/mglia-nat25).

For manuscripts utilizing custom algorithms or software that are central to the research but not yet described in published literature, software must be made available to editors and reviewers. We strongly encourage code deposition in a community repository (e.g. GitHub). See the Nature Portfolio guidelines for submitting code & software for further information.

Data

Policy information about availability of data

All manuscripts must include a data availability statement. This statement should provide the following information, where applicable:

- Accession codes, unique identifiers, or web links for publicly available datasets
- A description of any restrictions on data availability
- For clinical datasets or third party data, please ensure that the statement adheres to our policy

All raw sequencing data will be deposited and publically accessible on Gene Expression Omnibus (GSE296073 and GSE274829), NCBI.
All other data are available upon request.

Research involving human participants, their data, or biological material

Policy information about studies with human participants or human data. See also policy information about sex, gender (identity/presentation), and sexual orientation and race, ethnicity and racism.

Reporting on sex and gender	De-identified human specimens from both males and females were applied in this study, see more details in extended table 1.
Reporting on race, ethnicity, or other socially relevant groupings	N/A
Population characteristics	Postmortem human specimens from gestinational week 15 to postnatal week 3 have been used in this study.
Recruitment	De-identified human specimens were collected from the Autopsy Service in the Department of Pathology at the University of California San Francisco (UCSF) (Extended Data table 2), with previous patient consent in strict observance of the legal and institutional ethical regulations. The autopsy consents and all protocols for human prenatal brain tissue procurement were approved by the Human Gamete, Embryo and Stem Cell Research Committee (Institutional Review Board GESCR# 10- 02693) at UCSF.
Ethics oversight	UCSF: Institutional Review Board GESCR# 10- 02693

Note that full information on the approval of the study protocol must also be provided in the manuscript.

Field-specific reporting

Please select the one below that is the best fit for your research. If you are not sure, read the appropriate sections before making your selection.

☒ Life sciences ☐ Behavioural & social sciences ☐ Ecological, evolutionary & environmental sciences

For a reference copy of the document with all sections, see nature.com/documents/nr-reporting-summary-flat.pdf

Life sciences study design

All studies must disclose on these points even when the disclosure is negative.

Sample size	No methods were used to predetermine sample sizes. Regarding to experiments using postmortem human tissues, we included as many samples per age range as we can obtain. For others, minimum sample sizes were determined based on previously published studies.<br>For snRNAseq, donors=6;<br>For scRNAseq of MGEO, two batch of experiments were conducted. Each batch contains 12-16 organoids in each condition.<br>For scRNAseq of iMG in organoids, 16 6-week-old MGEOs with iMG were used for iMG library preparation.<br>For IHC with postmortem human tissues, generally 3-6 de-identified samples were used for each experiment.<br>For IHC with MGEO and Cortical organoids, 3-11 organoids were used for each used. No statistical methods were used to predetermine sample sizes.<br>For IHC with mouse tissues, 5-7 fetuses or pups from 3 litters were used for each experiment.<br>For RT-qPCR confirmation of IGF1 KO cell line, 3 biological repeats were applied.
Data exclusions	no data were excluded
Replication	For scRNAseq of MGEO to test the effect of iMG on MGEO development, two batches of organoids were used. Each batch has 12-16 organoids in each condition.<br>For IHC to test the effect of H9 hESC-induced iMG, IGF1 treatment, IGF1R inhibitor treatment, and IGF1 KO on 1323-4 hiPSC-induced MGEO development, 3-4 batches of organoids were used. For IHC to test microglia distribution in MGEOs and DAPT treatment, two batches of organoids were used. For IHC to confirm the effect in other cell lines, at least 2 batches of organoids were tested.<br>For IHC in mouse experiments, three independent litters were tested.

	The biological repeats number "N" values (<10) were indicated by displaying individual data points in the figure and were also included in the figure legend.
Randomization	Organoids were randomized to each experimental groups. For other experiments, no covariates were considered since no treatment was administered.
Blinding	For all quantification, data were acquired and quantified blindly to genotype or treatment.

Reporting for specific materials, systems and methods

We require information from authors about some types of materials, experimental systems and methods used in many studies. Here, indicate whether each material, system or method listed is relevant to your study. If you are not sure if a list item applies to your research, read the appropriate section before selecting a response.

Materials & experimental systems

n/a	Involved in the study
☐	☒ Antibodies
☐	☒ Eukaryotic cell lines
☒	☐ Palaeontology and archaeology
☐	☒ Animals and other organisms
☒	☐ Clinical data
☒	☐ Dual use research of concern
☒	☐ Plants

Methods

n/a	Involved in the study
☒	☐ ChIP-seq
☐	☒ Flow cytometry
☒	☐ MRI-based neuroimaging

Antibodies

Antibodies used	Doublecortin (DCX) Rabbit 1:500 Cell Signaling Technology 4604S<br>Doublecortin (DCX) Guinea pig 1:500 EMD Millipore AB2253<br>Iba1 Guinea pig 1:500 Synaptic Systems 234 308<br>IGF1 Rat 1:250 R&D Systems MAB2913<br>IGF1 Goat 1:250 R&D Systems AF791<br>IGF1R Goat 1:100 R&D Systems AF-305-NA<br>Ki-67 Mouse 1:500 BD Pharmingen 550609<br>Ki-67 Rat 1:200 Invitrogen 14-5698-80<br>BrdU Mouse 1:50 - 1:100 BD Biosciences 347580<br>P2RY12 Rabbit 1:500 AnaSpec, Inc. AS-55043A<br>SOX2 Mouse 1:500 Santa Cruz Biotechnology sc-365823<br>NESTIN Mouse 1:100 BD Transduction Laboratories 611658<br>NESTIN Mouse 1:500 Millipore MAB5326<br>NKX2.1 Rabbit 1:500 Abcam ab76013<br>DLX2 Rabbit 1:250 Abcam ab272902<br>LHX6 Mouse 1:500 Santa Cruz Biotechnology sc-271433<br>GAD67 Mouse 1:250 Chemicon International MAB5406<br>NeuN Guinea pig 1:200 EMD Millipore ABN90<br>SST Rat 1:200 EMD Millipore MAB354<br>PV Mouse 1:250 EMD Millipore MAB1572<br>PAX6 Rabbit 1:250 Cell Signaling Technology 60433S<br>OLIG2 Rabbit 1:2500 Abcam ab225100<br>PU.1 Rabbit 1:100 Cell Signaling Technology 81886S
Validation	All of these antibodies are selected from published literature and the species and application were validated by the manufacturer.

Eukaryotic cell lines

Policy information about cell lines and Sex and Gender in Research

Cell line source(s)	The eWT-1323-4 hiPSC line 45 (female, RRID: CVCL_0G84) was obtained from the Conklin Laboratory (University of California, San Francisco (UCSF). WA09/H9 (female, RRID: CVCL_9773, NIH registration number: NIHhESC-10_0062) and WA01/H1 (male, RRID: CVCL_9771, NIH registration number: NIHhESC-10-0043) were obtained from the WiCell Research Institute (Madison, WI, USA). The NKX2.1-GFP cell line (female) was obtained from MCRI and Monash University (Parkville, Victoria, Australia).
Authentication	The cell lines were not authenticated since obtained.
Mycoplasma contamination	All stem cell lines were tested negative for mycoplasma.

Commonly misidentified lines (See ICLAC register) | No

Animals and other research organisms

Policy information about studies involving animals; ARRIVE guidelines recommended for reporting animal research, and Sex and Gender in Research

Laboratory animals	C57/B6 mice, age E12.5 to P5; both male and female were used in this study.<br>2-6 months male and female IGF1 f/f mice (Jax, 012663) and 2-6 months male and female Cx3cr1-CreERt/+ (Jax, 020940) mice were crossed to generate IGF1f/f, Cx3cr1-CreERt/+ (F2) mice for this study. E14.5 IGF1f/f, Cx3cr1-CreERt/+ fetuses and their littermates were involved in this study. Both male and female were involved in this study.
Wild animals	No wild animals were used in this study.
Reporting on sex	both embryonic male and female were used in this study.
Field-collected samples	No field collected samples were used in the study.
Ethics oversight	All mice were handled according to the guidelines of the Institutional Animal Care and Use Committee at the University of California, San Francisco.

Note that full information on the approval of the study protocol must also be provided in the manuscript.

Plants

Seed stocks	*Report on the source of all seed stocks or other plant material used. If applicable, state the seed stock centre and catalogue number. If plant specimens were collected from the field, describe the collection location, date and sampling procedures.*
Novel plant genotypes	*Describe the methods by which all novel plant genotypes were produced. This includes those generated by transgenic approaches, gene editing, chemical/radiation-based mutagenesis and hybridization. For transgenic lines, describe the transformation method, the number of independent lines analyzed and the generation upon which experiments were performed. For gene-edited lines, describe the editor used, the endogenous sequence targeted for editing, the targeting guide RNA sequence (if applicable) and how the editor was applied.*
Authentication	*Describe any authentication procedures for each seed stock used or novel genotype generated. Describe any experiments used to assess the effect of a mutation and, where applicable, how potential secondary effects (e.g. second site T-DNA insertions, mosiacism, off-target gene editing) were examined.*

Flow Cytometry

Plots

Confirm that:

☒ The axis labels state the marker and fluorochrome used (e.g. CD4-FITC).

☒ The axis scales are clearly visible. Include numbers along axes only for bottom left plot of group (a 'group' is an analysis of identical markers).

☒ All plots are contour plots with outliers or pseudocolor plots.

☒ A numerical value for number of cells or percentage (with statistics) is provided.

Methodology

Sample preparation	Described in Methods
Instrument	BD FACS Aria II cell sorter
Software	FACSDiva, Floreada.io
Cell population abundance	As shown in Figure 2b.
Gating strategy	FSC/SSC were used to identify single cell/single nuclei population. DAPI was used to identify single nuclei in single nuclei isolation. DAPI negative was used to identify live cells in single cell sorting. PU.1-PE, OLIG2-AF647, and GFP was used to identify selected population. Further sequencing results (Fig. 2c, Fig. 3l, extended data Fig. 3, extended data Fig. 9) validate the flow cytometry results.

☒ Tick this box to confirm that a figure exemplifying the gating strategy is provided in the Supplementary Information.
