# Supplementary material for: Microglia regulate GABAergic neurogenesis in prenatal human brain through IGF1
Source: Nature. 2025 Aug 6;646(8085):676–86. doi: 10.1038/s41586-025-09362-8 (PMC12527950; doi:10.1038/s41586-025-09362-8)
Supplement: Supplementary file 3 — Supplementary Tables 1–3. [file 41586_2025_9362_MOESM3_ESM.pdf]

Supplementary table 1 | The list of postmortem human samples used in snRNAseq

Case no.	sex	Region	Enrichment			Age (weeks)	Developing stages
			no enrichment	PU.1^{+}	OLIG2^{+}		
4	Female	Whole	Yes	Yes	Yes	GW22	embryonic
5	Female	Whole	Yes	Yes	Yes	GW23	embryonic
6	Female	Whole	Yes	No	No	GW23	embryonic
15	Female	Periventricle	Yes	Yes	Yes	GW 30	embryonic
15	Female	Cortex	Yes	Yes	Yes	GW 30	embryonic
20	Male	Periventricle	Yes	Yes	Yes	PW 2	perinatal
20	Male	Cortex	Yes	Yes	Yes	PW 2	perinatal
21	Male	Cortex	Yes	Yes	Yes	PW 3	perinatal
21	Male	Periventricle	Yes	Yes	Yes	PW 3	perinatal

Supplementary table 2 | Clinical and experimental demographics of collected human specimens

case no.	Age	Gender	brain region	Clinical History	Neuropathological diagnosis
1	GW15	U	left hemisphere	Cervical insufficiency	control
2	GW17	U	posterior	path cervical incompetence	control
3	GW17	U		elective termination	control
4	GW22	female	a whole hemisphere	elective termination	control
5	GW23	female	a whole hemisphere	elective termination	control
6	GW23	female	a whole hemisphere	elective termination	control
7	GW21-1/7	female	left hemisphere	elective termination	control
8	GW22			elective termination	control
9	GW22-6/7	male	right hemisphere, coronal	elective termination	control
10	GW23-3/7	male	periventricle, coronal	elective termination	control
11	GW22-1/7	male	periventricle, coronal	elective termination	control
12	GW23-1/7	Female	left hemisphere, periventricle and cortex	elective termination	control
13	GW23-3/7		left hemisphere	elective termination	control
14	GW23-3/7	Male	left hemisphere, coronal	elective termination	control
15	GW30	female	periventricle and superior frontal gyrus	biventricular heart failure	control
16	38GW+2week	Male	periventricle	pulmonary hypertension ; FoxF1 mutation (tx factor in endoth cells)	control
17	36Gw+3week		periventricle	hypoplastic left heart syndrome	control
18	GW39-1/7	Female	periventricle	respiratory failure, pulmonary arterial hypertension, acute renal failure	control
19	GW40	male	frontal cortex	hernia	control
20	GW39-1/7+2week	male	left hemisphere	Congenital disaphragmatic hernia (CDH)	control
21	GW40+3week	male	periventricle and temporal cortex	Consequence of low output state	control

Supplementary table 3 | List of antibodies used in IHC

Primary Ab	Species	Dilution	Manufacturer	Cat No.
Doublecortin (DCX)	Rabbit	1:500	Cell Signaling Technology	4604S
Doublecortin (DCX)	Guinea pig	1:500	EMD Millipore	AB2253
IBA1	Guinea pig	1:500	Synaptic Systems	234 308
IGF1	Rat	1:250	R&D Systems	MAB2913
IGF1	Goat	1:200	R&D Systems	AF791
IGF1R	Goat	1:100	R&D Systems	AF-305-NA
Ki-67	Mouse	1:500	BD Pharmingen	550609
Ki-67	Rat	1:200	Invitrogen	14-5698-80
BrdU	Mouse	1:50 - 1:100	BD Biosciences	347580
P2RY12	Rabbit	1:500	AnaSpec, Inc.	AS-55043A
SOX2	Mouse	1:500	Santa Cruz Biotechnology	sc-365823
NESTIN	Mouse	1:100	BD Biosciences	611658
NESTIN	Mouse	1:500	Millipore	MAB5326
NKX2.1	Rabbit	1:250-1:500	Abcam	ab76013
DLX2	Rabbit	1:250	Abcam	ab272902
LHX6	Mouse	1:500	Santa Cruz Biotechnology	sc-271433
GAD67	Mouse	1:250	Chemicon International	MAB5406
NeuN	Guinea pig	1:200	EMD Millipore	ABN90
SST	Rat	1:200	EMD Millipore	MAB354
PV	Mouse	1:250	EMD Millipore	MAB1572
PAX6	Rabbit	1:250	Cell Signaling Technology	60433S
